# Interaction Between the SNARE SYP121 and the Plasma Membrane Aquaporin PIP2;7 Involves Different Protein Domains

**DOI:** 10.3389/fpls.2020.631643

**Published:** 2021-01-18

**Authors:** Timothée Laloux, Irwin Matyjaszczyk, Simon Beaudelot, Charles Hachez, François Chaumont

**Affiliations:** Louvain Institute of Biomolecular Science and Technology, UCLouvain, Louvain-la-Neuve, Belgium

**Keywords:** aquaporin, syntaxin, interaction motif, SNAREs, plasma membrane intrinsic protein, ratiometric bimolecular fluorescence complementation

## Abstract

Plasma membrane intrinsic proteins (PIPs) are channels facilitating the passive diffusion of water and small solutes. Arabidopsis PIP2;7 trafficking occurs through physical interaction with SNARE proteins including the syntaxin SYP121, a plasma membrane Qa-SNARE involved in membrane fusion. To better understand the interaction mechanism, we aimed at identifying the interaction motifs in SYP121 and PIP2;7 using ratiometric bimolecular fluorescence complementation assays in *Nicotiana benthamiana*. SYP121 consists of four regions, N, H, Q, and C, and sequential deletions revealed that the C region, containing the transmembrane domain, as well as the H and Q regions, containing the Habc and Qa-SNARE functional domains, interact with PIP2;7. Neither the linker between the Habc and the Qa-SNARE domains nor the H or Q regions alone could fully restore the interaction with PIP2;7, suggesting that the interacting motif depends on the conformation taken by the HQ region. When investigating the interacting motif(s) in PIP2;7, we observed that deletion of the cytosolic N- and/or C- terminus led to a significant decrease in the interaction with SYP121. Shorter deletions revealed that at the N-terminal amino acid residues 18–26 were involved in the interaction. Domain swapping experiments between PIP2;7 and PIP2;6, a PIP isoform that does not interact with SYP121, showed that PIP2;7 N-terminal part up to the loop C was required to restore the full interaction signal, suggesting that, as it is the case for SYP121, the interaction motif(s) in PIP2;7 depend on the protein conformation. Finally, we also showed that PIP2;7 physically interacted with other Arabidopsis SYP1s and SYP121 orthologs.

## Introduction

Plasma membrane intrinsic proteins (PIPs) are aquaporins facilitating the diffusion of water and other small solutes across the plasma membrane. They are involved in a diversity of plant physiological processes including cell expansion and water homeostasis, regulation of root and leaf hydraulic conductivity as well as in photosynthesis (Chaumont and Tyerman, 2014). Aquaporins consist of six transmembrane domains (TM) linked by five loops, the N- and C-termini facing the cytosol. As membrane integral proteins, they are synthesized and co-translationally inserted in the endoplasmic reticulum (ER) membrane and travel across the secretory pathway to reach their target membrane.

Recent studies have highlighted the role of SNAREs (soluble N-ethylmaleimide-sensitive factor attachment protein receptors) in the subcellular trafficking and regulation of the PIP aquaporins (Besserer et al., 2012; Hachez et al., 2014). SNAREs are membrane proteins mainly involved in the addressing and fusion of vesicles with their target membranes. Each compartment having their own resident SNAREs, recognition between SNAREs located on the vesicles and those located on the membranes, together with tethering factors and other regulatory proteins drives correct vesicle addressing and fusion to the target membrane. About 65 members of the SNARE family have been identified in Arabidopsis, two times more than in unicellular or mammalian organisms, suggesting an important role of these proteins in a complex endomembrane system including distinct secretory and vacuolar trafficking events (Sanderfoot, 2007; Kim and Brandizzi, 2012). They were classified on the basis of the SNARE domain structure: the R-SNAREs have an arginine residue (R) in the center of the SNARE domain, while Q-SNAREs have a glutamine residue (Q) (Fasshauer et al., 1998). SNAREs drive vesicle fusion and membrane intercalation by assembling in ternary complexes of cognate partner Qa-, Qb-, Qc-, and R-SNAREs (Lipka et al., 2007). The Qa-SNAREs possess a long N-terminal region containing a Habc domain made of three short helices Ha, Hb, Hc that mimic the structure of the SNARE (Lipka et al., 2007). Among them, the Syntaxin of Plant (SYP) 1s are Qa-SNARE localized at the plasma membrane (Uemura et al., 2004; Enami et al., 2009). SYP121, one of the nine SYP1s found in Arabidopsis, is involved in the fusion of vesicles with the plasma membrane, together with its cognate SNARE Synaptosome-Associated Protein (SNAP) 33, a Qb+Qc-SNARE, and the R-SNAREs Vesicle-Associated Membrane Protein (VAMP) 721 and 722 (Kargul et al., 2001; Kwon et al., 2008; Kwaaitaal et al., 2010). It has the typical structure of Qa-SNAREs with, in front of the Habc, a 39-residue N-terminal region (Sanderfoot, 2007; Tyrrell et al., 2007; Grefen et al., 2010; Fujiwara et al., 2014). SYP121 is expressed more abundantly in the epidermal cells and lateral root cap cells of the root tip, this signal decreasing with root growth. However, it is expressed in all tissues, albeit at lower levels (Enami et al., 2009).

In *Zea mays* and *Arabidopsis thaliana*, SYP121 isoforms regulate the proper trafficking of ZmPIP2;5 and AtPIP2;7 through physical interactions. The expression of SYP121ΔC (previously name Sp2 fragment), a truncated version that does not contain the C-terminal TM and behaves as a dominant negative mutated protein, reduces the accumulation of AtPIP2;7 and ZmPIP2;5 in the plasma membrane (Besserer et al., 2012; Hachez et al., 2014). This effect is specific for SYP121 as the expression of the ΔC deletions of the plasma membrane-localized SYP122 and the Qc-SNARE SYP71 as well as the prevacuolar compartment Qa-SNARE AtSYP21 does not alter ZmPIP2;5 trafficking (Besserer et al., 2012). Consequently, the membrane osmotic water permeability coefficient (P_f_) of protoplasts co-expressing SYP121ΔC with the PIPs is reduced compared with the P_f_ of protoplasts expressing the PIPs alone (Besserer et al., 2012; Hachez et al., 2014). The expression of ZmSYP121ΔC also reduces the P_f_ of Xenopus oocytes co-expressing ZmPIP2;5 compared with the oocytes expressing ZmPIP2;5 alone, but not ZmPIP2;5 plasma membrane abundance, suggesting that SYP121 regulates not only PIPs trafficking but also their gating (Besserer et al., 2012; Hachez et al., 2014). The results collected on these distant plant species suggest that this interaction predates the divergence between monocots and dicots about 200 million years ago.

In addition to the regulation of PIP aquaporins, SYP121 also physically interacts with K^+^ channels to regulate their trafficking to the plasma membrane (Sutter et al., 2006), and the KC1/AKT1 heterotetramer activity. Indeed, when SYP121 is co-expressed with AKT1/KC1 in oocytes, the negative shift of the voltage threshold is reduced, therefore promoting the opening of the channel (Honsbein et al., 2009; Grefen et al., 2010). This interaction is mediated by an FxRF motif located within the first 12 N-terminal residues of SYP121 (Grefen et al., 2010). KC1 also binds to SYP121 through a conserved RYxxWE motif located at the cytosolic face of the voltage sensing domain (Grefen et al., 2015). The same motif is involved in the interaction between KAT1 and SYP121, leading to the same impact on the activation of KAT1 (Lefoulon et al., 2018). Conversely to the role of the complex in K^+^ uptake, *akt1*, *kc1*, and *syp121* mutated lines share the same impaired growth phenotype in rate-limiting concentrations of K^+^ (Honsbein et al., 2009). Moreover, the tobacco homolog of SYP121, NtSYR1, is involved in the K^+^, Cl^–^, and Ca^++^ channel activity in guard cells of the stomatal complexes. Over-expression of the SYP121ΔC fragment blocks the response of the channels to abscisic acid and therefore the response of plants to a lack of water (Leyman et al., 1999; Sokolovski et al., 2008).

Altogether these results led to consider SYP121 as a “super-coordinator” of plant cellular homeostasis and cell expansion (Grefen and Blatt, 2008; Besserer et al., 2012; Karnik et al., 2017). These processes depend on the turgor pressure resulting from the uptake and accumulation of inorganic ions and water. SYP121, by controlling aquaporin and K^+^ channel abundance and/or activity in the plasma membrane, might act as a coordinator of ion and water uptake to regulate cell expansion or swelling, through the possible formation of a PIP/SYP121/K^+^ channel tripartite. To better understand this regulation mechanisms, identification of the interaction motifs between PIPs and SYP121 was a first step to further modify/disrupt the SYP121/PIP complex association and analyze the physiological consequences *in planta*. To this aim, we tested series of deletions and mutations in SYP121 and PIP2;7 using ratiometric bimolecular fluorescence complementation assays in *Nicotiana benthamiana*, and showed that the interaction between both proteins involved several domains suggesting the importance of the protein conformation in this interaction.

## Materials and Methods

### Molecular Cloning

Cloning was carried out using the Gateway technology (Thermo Fisher). Briefly, the full-length cDNAs were amplified by PCR with a couple of primers (Supplementary Table 1) harboring either the *attB1, attB2, attB3*, or *attB4* sequence, from either total cDNA (extracted from 7-d-old seedlings) or vectors already available in the lab. After purification, the PCR products were inserted into a donor vector through BP cloning thanks the BP clonase II enzyme kit. The resulting entry vectors were verified by restrictions and sequencing. Transfer of the Gateway cassette from the entry vectors to the destination vector was performed through LR clonase II mediated recombination. Both BP and LR cloning were done according to the manufacturer’s recommendations. The resulting expression plasmids were verified by restriction and, in some cases, by PCR or sequencing.

The pBiFCt-2in1 vectors (Grefen and Blatt, 2012) were used to carry out rBiFC assays. The cDNAs were subcloned in pDONR221-P3P2 or pDONR221-P1P4 entry vectors prior to their integration in the pBiFCt-2in1 vectors following the authors’ recommendations. The 2in1-BiFCt-NN vector was mostly used for the production of proteins fused at their N-terminal end to the split Enhanced yellow fluorescent protein (EYFP) fragments. The cDNAs inserted in the pDONR221-P3P2 were translated in fusion with the nEYFP, while the cDNAs inserted in the pDONR221-P1P4 were translated in fusion with the cEYFP. The *PIP* cDNAs were inserted in the vector pDONR221-P1P4, while the *SNARE* cDNAs were inserted in the vector pDONR221-P3P2. Therefore, in the final pBiFCt-2in1 vector, the *PIP* cDNAs were fused to the sequence encoding the cEYFP fragment while the *SNARE* cDNAs were fused to the sequence encoding the nEYFP fragment. The pFRETtv-2in1-NN vector (Hecker et al., 2015) was used to determine the subcellular localization of the proteins. The same protocol as for the pBiFCt-2in1 vectors was followed for the plasmid generation.

### Plant Growth

*N. benthamiana* seeds were germinated and the plants grown in a phytotron [8 h dark/16 h light regime at 25°C (day)/18°C (night) temperature with a light intensity of approximately 200 μmol photon m^–2^ s^–2^].

### *Agrobacterium* Infiltration

The plasmids were transiently expressed in *N. benthamiana* leaves through agro-infiltration with the *Agrobacterium tumefaciens* strain AGL1 (Lazo et al., 1991) according to Hachez et al. (2014). Electrocompetent AGL1 cells were transformed using 500 ng of the DNA minipreparation.

For the infiltration, colonies were incubated with agitation overnight at 28°C in 5 ml LB containing the appropriate antibiotic. The cells were pelleted by centrifugation at 5,000 *g* for 5 min at Room Temperature (RT), washed twice with 2 mL of the infiltration buffer [50 mM MES, 2 mM Na_3_PO_4_, 0.5% (w/v) glucose, pH 5.6] and resuspended in 2 mL of the same buffer supplemented with 100 mM acetosyringone. The bacterial suspension was then incubated for 2 h at RT in the dark to activate the virulence. The inoculum was delivered to *N. benthamiana* leaves by gentle pressure infiltration through the stomata of the abaxial side, using a 1 mL syringe without a needle. Samples were analyzed 3-day post-infiltration to allow sufficient time for protein production.

### Confocal Microscopy

Plant materials were imaged according to standard procedures on a Zeiss LSM710 confocal microscope equipped with a spectral detector. The confocal setting used for the rBiFC experiments were adapted from the one used in Grefen and Blatt (2012) according to the modules installed on the LSM710. The acquisitions of the rBiFC data were performed with two different microscope settings. The excitation wavelength (laser intensity)/emission bandwidth/dichroic filter/master gain settings were the following: first settings, Yellow fluorescent protein (YFP), 514 nm (10%)/BP 522–553/MBS [458/514]/797; Red fluorescent protein (RFP), 561 nm (5%)/BP 559–615/MBS [488/561/633]/795; second settings, YFP, 514 nm (10%)/BP 522–553/MBS [458/514/561/633]/795; RFP, 561 nm (7%)/BP 559–615/MBS [458/514/561/633]/780.

### Ratiometric BiFC Assays

The BiFC assay developed by Grefen and Blatt (2012) allows a ratiometric quantification of the interaction by comparing the fluorescence intensity level of an internal control (RFP), and the reconstituted YFP signal. As all the cDNAs were inserted in the same vector and their expression driven by the same p35S promoter, a theoretically equal production rate is obtained for the comparison of the fluorescence. The fluorescence of each cell was quantified using three different 4 μm lines along the plasma membrane. The YFP/RFP ratio of each line was calculated based on the maximum intensities of both RFP and YFP signals along the lines. The mean of the three ratios was used for statistical analysis. At least 10 cells were analyzed per pairs in at least two repetitions. The positive (SYP121/PIP2;7) and negative (SNAP33/PIP2;7) controls were infiltrated in each experiment. For statistical analysis, we considered not only the ratios obtained for each pair but also the impact of the repetitions. All the experiments done using the same setting were pulled together and the results compared with the respective positive and negative controls to evaluate the effect of the interacting pair on the YFP/RFP ratio. Therefore, a linear mixed model, with a random intercept taking the repetition’s dependency into account was fitted using SAS software (SAS Institute Inc, 2016). Based on the model estimates, mean comparisons were proceeded using Dunnett’s test. Graphs were created using the R software (R Core Team, 2019) and the ggplot2 package (Wickman, 2009).

## Results

### Different Parts of SYP121 Are Required for Its Interaction With PIP2;7

**FIGURE 1:**
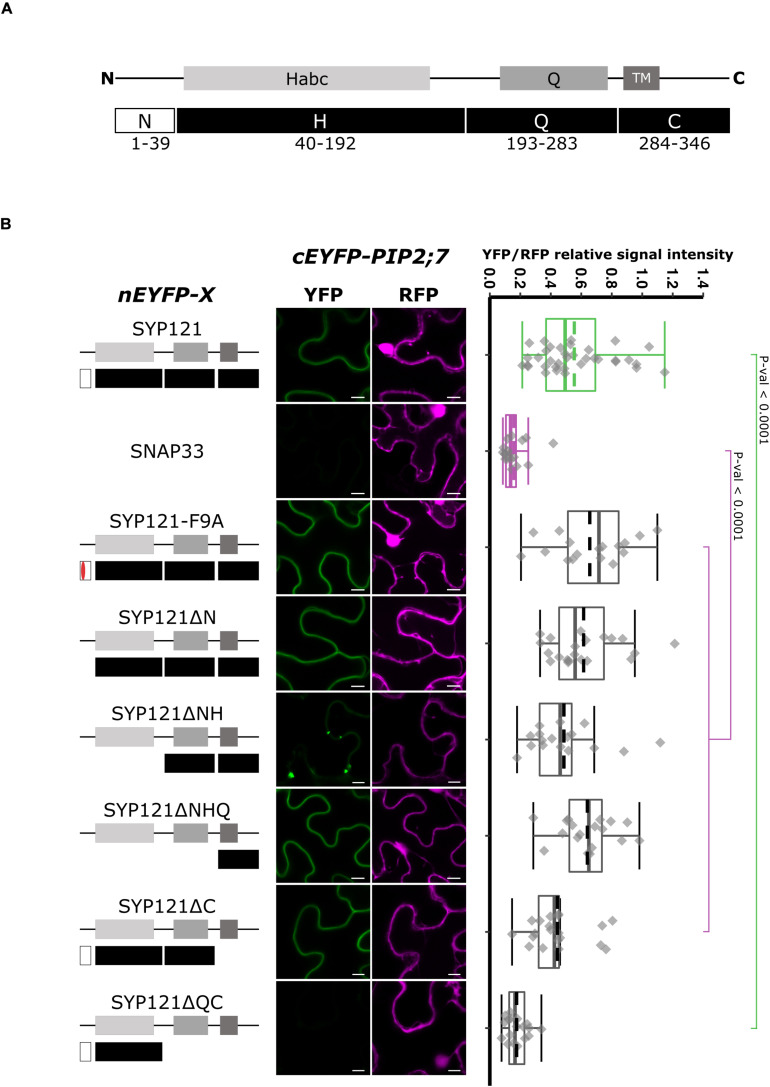
rBiFC assays for the interaction between nEYFP-SYP121 deletions and cEYFP-PIP2;7. **(A)** SYP121 topology. SYP121 N-terminus region is 39 amino acid residue long and contains the FxRF motif located at positions 9–12. The H region of SYP121 (residues 44–192) contains the regulatory domain Habc. The Q region (residues 193–283) contains the Qa-SNARE motif and is followed by the C-terminus region (residues 284–346) containing the TM and the extracellular extremity [adapted from Grefen et al. (2010)]. The size of the deletions is not representative. **(B) On the left:** nEYFP-SYP121 fusion topology. The mutated FxRF motif is in red. **Center panel:** representative rBiFC images. Images on the left show the YFP signal resulting from protein interaction, while those on the right show the control RFP signal. The scale bar represents 10 μm. **On the right:** ratiometric quantification of the fluorescent signals. Between 17 and 21 cells for each protein pair were analyzed by pair minus the positive and negative controls (see “Materials and Methods”). The positive control nEYFP-SYP121/cEYFP-PIP2;7 and negative control nEYFP-SNAP33/cEYFP-PIP2;7 were colored in green and purple, respectively. SYP121 and SNAP33 rBiFC ratios were always significantly different (*p*-value < 0.0001). This was not indicated to avoid overcharging the graph. In the box plots, the center lines of boxes represent the medians with outer limits at 25th and 75th percentiles. Dashed lines represent means. Tukey whiskers extend to 1.5 interquartile range.

Syntaxin proteins can be dissected in four regions: N, H, Q, and C (Figure 1A). The N region (N-terminus) contains the FxRF motif required for SYP121 interaction with KC1 (Grefen et al., 2010), the H region contains the regulatory domain Habc, the Q region contains the Q-SNARE motif, and the C region (C-terminus) contains a TM domain (Figure 1A). Several genetic constructs encoding deletions starting from either the C- or the N-terminus of SYP121 were prepared to identify the region required for the interaction with PIP2;7, using rBiFC assays. In all the rBiFC experiments included in this work, the proteins were expressed fused at the N-terminus with the n or cEYFP fragments because it is the only extremity facing the cytosol for the SNAREs and this fusion configuration does not to interfere with the trafficking and function of PIP2;7 or of the other PIPs (Fetter et al., 2004; Zelazny et al., 2007, 2009; Hachez et al., 2014). In addition, the SYP121/PIP2;7 and SNAP33/PIP2;7 pairs were used as positive and negative controls, respectively (Hachez et al., 2014). SNAP33 is a Qb+ Qc-SNARE belonging to the SNARE complex of SYP121 (Kwaaitaal et al., 2010). The resulting fluorescent signals were systematically quantified for comparison (see “Materials and Methods”). In the statistical analysis, both controls were always different with a *p*-value < 0.0001.

We first investigated whether the FxRF motif, which is involved in the interaction with KC1, also interacted with PIP2;7. To this end, we tested the F9A substitution (Grefen et al., 2010) and showed that this motif was not involved in the interaction with PIP2;7 (Figure 1B). We then checked if the interaction was retained with different deleted SYP121 proteins. An interaction with PIP2;7 in the plasma membrane was still detected for SYP121ΔN and SYP121ΔNHQ (Figure 1B). A weaker signal was observed for SYP121ΔNH in the plasma membrane, but strong signals labeled internal structures (Figure 1B and Supplementary Figure 1). These results suggested that the SYP121 C-terminal region was required for the interaction with PIP2;7. However, deletion of the C-terminal part (SYP121ΔC) gave rise to a fluorescent signal, which was absent when the Q and C regions were both deleted (SYP121ΔQC) (Figure 1B). Quantification of the YFP/RFP signal ratios confirmed the interaction data obtained between PIP2;7 and the SYP121 deletions (Figure 1B). These results might appear contradictory as both SYP121ΔC and SYP121ΔNHQ were interacting with PIP2;7. However, the fluorescence signal ratio for SYP121ΔC/PIP2;7 pair was lower than the other pair ratios of pairs giving a fluorescent signal, and was significantly different from the signal ratio obtained for SYP121-F9A and PIP2;7, suggesting that the SYP121 C-terminus was indeed important for the interaction with PIP2;7. In addition, more than one motif/region or a region overlapping the Q and C junction might also be required.

To verify that the absence of a fluorescent signal for SYP121ΔQC/PIP2;7 was not due to a problem of protein expression, we expressed all the SNARE versions tested in Figure 1 in fusion with the monomeric mTRQ2. As the proteins were expressed in similar conditions and under the control of the same promoter than in the BiFC experiments, the presence of a fluorescent signal could reflect the expression of the proteins in the rBiFC assays, A signal was observed for all the SYP121 deletions including the mTRQ2-SYP121ΔQC (Supplementary Figure 2A), suggesting the absence of interaction in rBiFC assays cannot be due to a lack of expression of the proteins.

**FIGURE 2:**
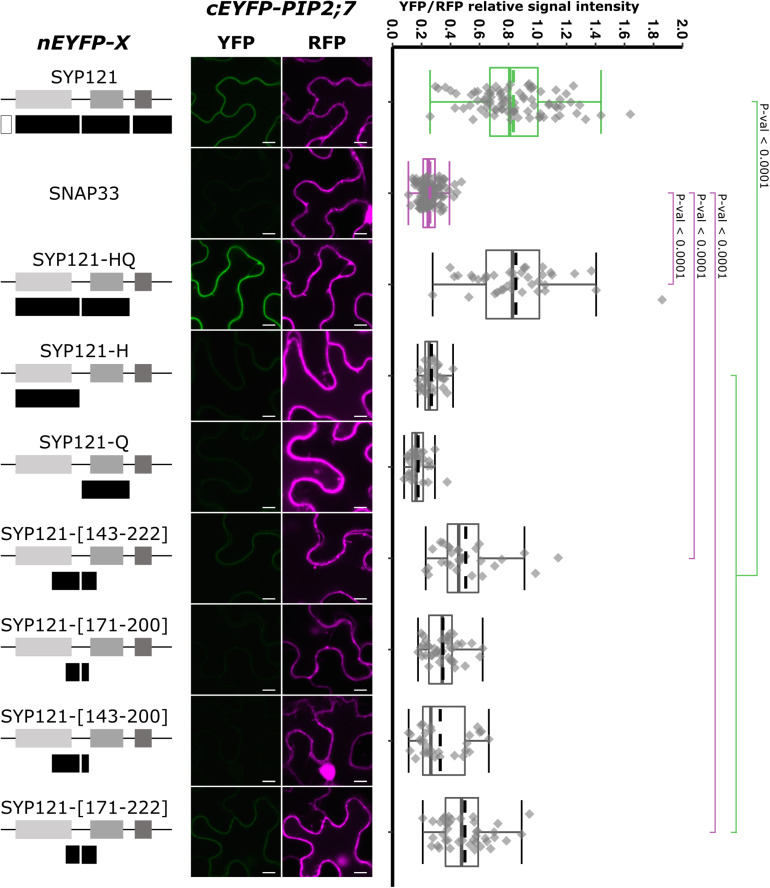
rBiFC assays for the interaction between nEYFP-SYP121 deletions at the HQ region and cEYFP-PIP2;7. **On the left:** nEYFP-SYP121 fusion topology. The size of the deletions is not representative. **Center panel:** representative rBiFC images. Images on the left show the YFP signal resulting from protein interaction, while those on the right show the control RFP. The scale bar represents 10 μm. **On the right:** ratiometric quantification of the fluorescent signals. Between 10 and 40 cells for each protein pair were analyzed as described in Figure 1.

We then tested the interaction between PIP2;7 and SYP121-HQ or either the H or the Q region alone (Figure 2). A positive interaction was observed for SYP121-HQ/PIP2;7 pair but not for SYP121-H/PIP2;7 or SYP121-Q/PIP2;7 pairs (Figure 2), suggesting a role of the H and Q overlapping region. We investigated the linker region between the Habc and Qa-SNARE domain, which includes the amino acid residues 171–200. Four constructs encoding SYP121-[143–222], SYP121-[171–200], SYP121-[143–200], and SYP121-[171–222] were then designed. Whereas no interaction with PIP2;7 was observed for SYP121-[171–200] and SYP121-[143–200], a low but significantly different fluorescence ratio was observed for SYP121-[143–222] and SYP121-[171–222] compared with negative control (Figure 2).

Altogether, the SYP121 deletions allowed us to show that (i) the FxRF motif, which interacts with KC1, was not involved in PIP2,7 interaction and (ii) two parts of SYP121 including the amino acid residues 171–222 of the HQ region and the C-terminus were involved in this interaction.

### PIP2;7 N- and C-Termini Are Required for PIP2;7/SYP121 Interaction

**FIGURE 3:**
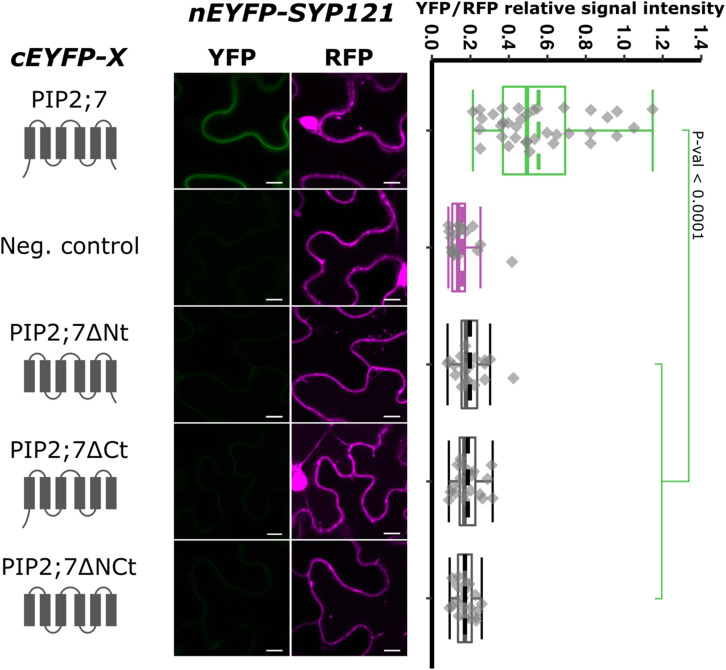
rBiFC assays for the interaction between cEYFP-PIP2;7 deletions and nEYFP-SYP121. **On the left:** cEYFP-PIP2;7 fusion topology. PIP2;7ΔNt is deleted from the first 35 amino acids, up to the beginning of the TM1. PIP2;7ΔCt is deleted from the last 18 amino acids starting at the end of the TM6. PIP2;7ΔNCt combines both deletions. The beginning and end of the TM1 and TM6 were selected by multiple alignments of plant PIP2 sequences. The negative control is SNAP33. **Center panel:** representative rBiFC images. Images on the left show the YFP signal resulting from protein interaction, while those on the right show the control RFP signal. The scale bar represents 10 μm. **On the right:** ratiometric quantification of the fluorescent signals. Twenty cells for each protein pair were analyzed as described in Figure 1.

Aquaporins are composed of six TMs connected by five loops, both the N- and C-termini being located in the cytosol. To identify the PIP2;7 motif that interacts with SYP121, we first analyzed the interaction between SYP121 and different truncated versions of PIP2;7 (Figure 3). Deletion of the N- or C-terminus resulted in the loss of interaction with SYP121, suggesting that both extremities are involved in the interaction. The interaction of PIP2;7ΔNt and PIP2;7ΔCt with SYP121ΔC, a soluble version of SYP121, was also tested to be sure that a possible modification in the subcellular localization of the deleted PIP2;7 versions or SYP121 was not the reason why the proteins did not interact. No interaction was observed between PIP2;7ΔNt and PIP2;7ΔCt with SYP121ΔC (Supplementary Figure 3). These proteins fused to mVenus were well expressed and localized in the plasma membrane and intracellular structures probably corresponding to the ER and Golgi apparatus (Supplementary Figure 2A).

**FIGURE 4:**
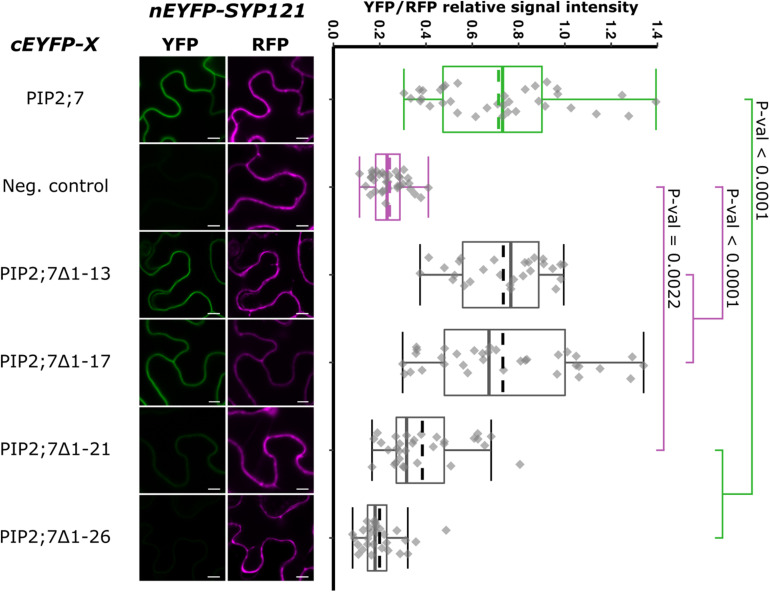
rBiFC assays for the interaction between cEYFP-PIP2;7 N-terminus deletions and nEYFP-SYP121. **On the left:** representative rBiFC images. Images on the left show the YFP signal resulting from protein interaction, while those on the right show the control RFP signal. The scale bar represents 10 μm. **On the right:** ratiometric quantification of the fluorescent signals. Between 27 and 34 cells for each protein pair were analyzed as described in Figure 1. The negative control is nEYFP-SNAP33/cEYFP-PIP2;7.

**FIGURE 5:**
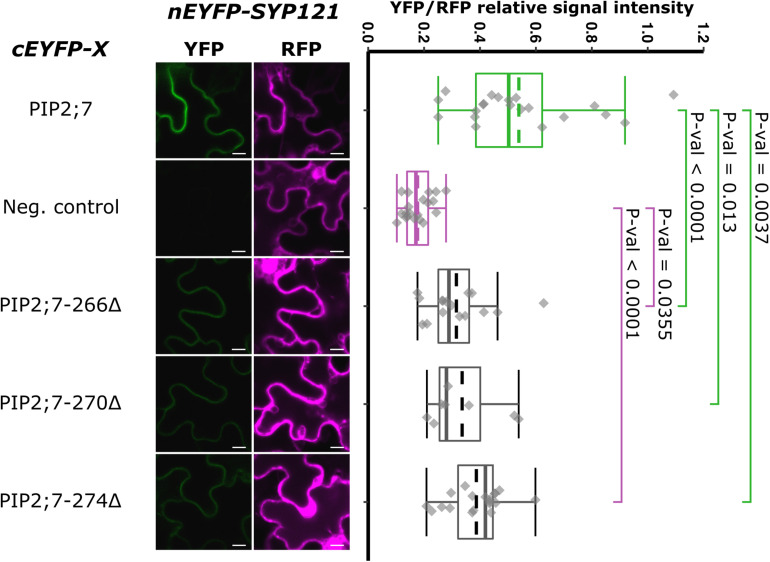
rBiFC assays for the interaction between cEYFP-PIP2;7 C-terminus deletions and nEYFP-SYP121. **On the left:** representative rBiFC images. Images on the left show the YFP signal resulting from protein interaction, while those on the right show the control RFP signal. The scale bar represents 10 μm. **On the right:** ratiometric quantification of the fluorescent signals. Between 10 and 19 cells for each protein pair were analyzed as described in Figure 1. The negative control is nEYFP-SNAP33/cEYFP-PIP2;7.

We then tested shorter regions of both the N- and C-termini. For the N-terminus, the first 13, 17, 21, and 26 amino acid residues were sequentially removed. A YFP signal was observed for the SYP121/PIP2;7Δ1–13 and SYP121/PIP2;7Δ1–17 pairs, while a weak or no signal was detected for the SYP121/PIP2;7Δ1–21 and SYP121/PIP2;7Δ1–26 pairs, respectively (Figure 4). We verified that mVenus-PIP2;7Δ1–21 was well expressed and the fluorescent signal was found in the plasma membrane and internal structures (Supplementary Figure 2A). The fluorescent YFP/RFP ratios of SYP121/PIP2;7Δ1–21 pair was different from both controls while SYP121/PIP2;7Δ1–26 was not different from the negative control (Figure 4). Deletions of the last 6, 10, and 14 amino acid residues of the PIP2;7 C-terminus were also tested for their interaction with SYP121 (Figure 5). A weak YFP signal was detected for all of them, the fluorescence ratios of the pairs being different from SYP121/PIP2;7. Both SYP121/PIP2;7–266Δ and SYP121/PIP2;7–274Δ but not SYP121/PIP2;7–270Δ ratios were different from the negative control (Figure 5). Altogether, these data indicate that the N-terminal amino acid residues 18–26 were required for the interaction with SYP121 and that the whole C-terminus play a role in the interaction, possibly by influencing the proper conformation of the N-terminus rather than containing a motif itself.

To locate more accurately the PIP2;7 interaction motif, we wanted to perform swapping experiments between PIP2;7 and another PIP2 that did not interact with SYP121. To identify such a PIP2, we screened the members of the Arabidopsis PIP2 subfamily, as well as PIP1;4 (Supplementary Figure 4). The only PIP protein that did not give rise to a fluorescent signal when co-expressed with SYP121 was PIP2;6. Quantification of the YFP/RFP fluorescence ratios showed that all the PIP/SYP121 pairs produced a significantly different ratio from the negative control (albeit with different *p*-values), with the exception of SYP121/PIP2;6. To verify that PIP2;6 was well expressed, we cloned PIP2;6 cDNA in the pFRETtv-2in1 vectors and detected the expression of mVenus-PIP2;6 in transient expression in *N. benthamiana* (Supplementary Figure 2B).

**FIGURE 6:**
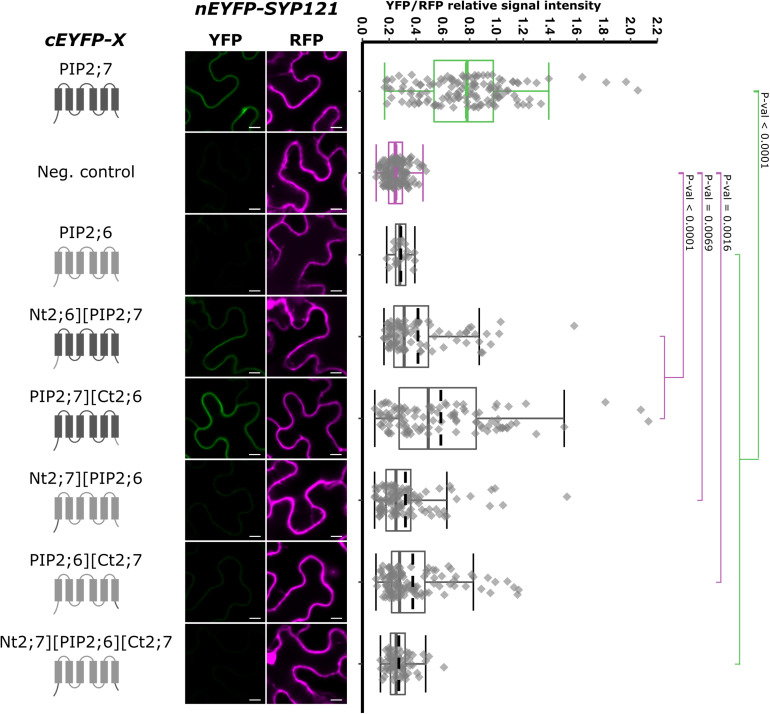
rBiFC assays for the interaction cEYFP-PIP2;7][PIP2;6 N- and C-terminus swapped proteins and nEYFP-SYP121. **On the left:** cEYFP-PIP fusion topology. PIP2;7 and PIP2;6 sequences are represented in dark and light gray, respectively. The beginning and end of the TMs and loops were selected by multiple alignments of plant PIP2 sequences. The negative control is SNAP33. **Center panel:** representative rBiFC images. Images on the left show the YFP signal resulting from protein interaction, while those on the right show the control RFP signal. The scale bar represents 10 μm. **On the right:** ratiometric quantification of the fluorescent signals. Between 70 and 120 cells for each protein pair were analyzed as described in Figure 1. The negative control is nEYFP-SNAP33/cEYFP-PIP2;7.

Using this non-interacting PIP, we performed swapping experiments, starting from the whole N- and/or C-termini and generated pBiFCt-2in1 plasmids containing SYP121 and the following chimeras: Nt2;6][PIP2;7, PIP2;7][Ct2;6, Nt2;7][PIP2;6, PIP2;6][Ct2;7, and Nt2;7][PIP2;6][Ct2;7 (Figure 6). Quite a large variation in the fluorescence intensity was obtained in the different rBiFC repetitions for the swapped constructs/SYP121 pairs, which was confirmed by the quantification of fluorescence ratios. All pairs gave significantly different ratios compared with the ratio of either the positive or the negative controls, with the exception of SYP121/Nt2;7][PIP2;6][Ct2;7 ratio, which was not different from the negative control ratio (Figure 6). The chimera giving the highest fluorescent signals compared to the other chimeras was PIP2;7][Ct2;6, suggesting that the (i) PIP2;7 N-terminus was required for the interaction with SYP121 and (ii) the C-terminus of PIP2;7 was not absolutely necessary or that the C-terminus of PIP2;6 could partly replace the PIP2;7 C-terminus. On the other hand, the absence of interaction between SYP121 and Nt2;7][PIP2;6][Ct2;7 was unexpected, but indicated that the N- and C-termini of PIP2;7 were not enough to restore an interaction of PIP2;6 with SYP121 and that other PIP2;7 domain(s) were required. This could explain why the SYP121/Nt2;6][PIP2;7, SYP121/Nt2;7][PIP2;6, and SYP121/PIP2;6][Ct2;7 pairs generated low ratios, although slightly different from the negative control.

**FIGURE 7:**
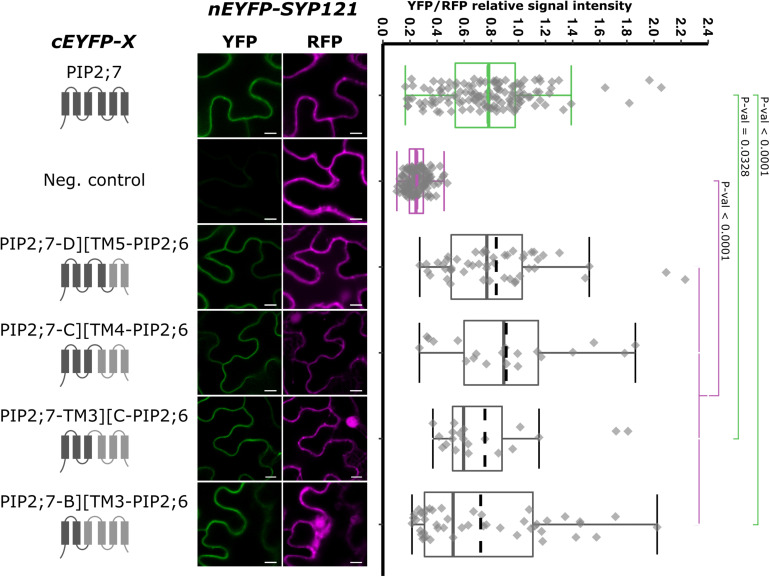
rBiFC assays for the interaction between cEYFP-PIP2;7][PIP2;6 loop and TM swapped proteins and nEYFP-SYP121. **On the left:** cEYFP-PIP fusion topology. PIP2;7 and PIP2;6 sequences are represented in dark and light gray, respectively. The beginning and end of the TMs and loops were selected by multiple alignments of plant PIP2 sequences. **Center panel:** representative rBiFC images. Images on the left show the YFP signal resulting from protein interaction while those on the right show the control RFP signal. The scale bar represents 10 μm. **On the right:** ratiometric quantification of the fluorescent signals. Between 20 and 50 cells for each protein pair were analyzed as described in Figure 1. The negative control is nEYFP-SNAP33/cEYFP-PIP2;7.

As we showed that the swapping of both PIP2;7 N- and C-termini in PIP2;6 was not sufficient for an interaction with SYP121, we generated chimeras including the TMs and loops. We generated pBiFCt-2in1 plasmids containing SYP121 together with PIP2;7-D][TM5-PIP2;6, PIP2;7-C][TM4-PIP2;6, PIP2;7-TM3][C-PIP2;6, and PIP2;7-B][TM3-PIP2;6, D, C, and B indicating that the PIP2;7 fragments in these fusion ended after the loop D, C, or B, respectively (Figure 7). Analysis of the fluorescent ratios showed that, while all of them were different from the negative control, only SYP121/PIP2;7-D][TM5-PIP2;6 and SYP121/PIP2;7-C][TM4-PIP2;6 ratios were not significantly different from the positive control (Figure 7). The lower fluorescence ratio obtained for PIP2;7-TM3][C-PIP2;6 and PIP2;7-B][TM3-PIP2;6 suggested that the PIP2;7 loop C was playing a role in the interaction.

### SYP1s, Except SYP112, and SYP121 Orthologs Interact With PIP2;7

**FIGURE 8:**
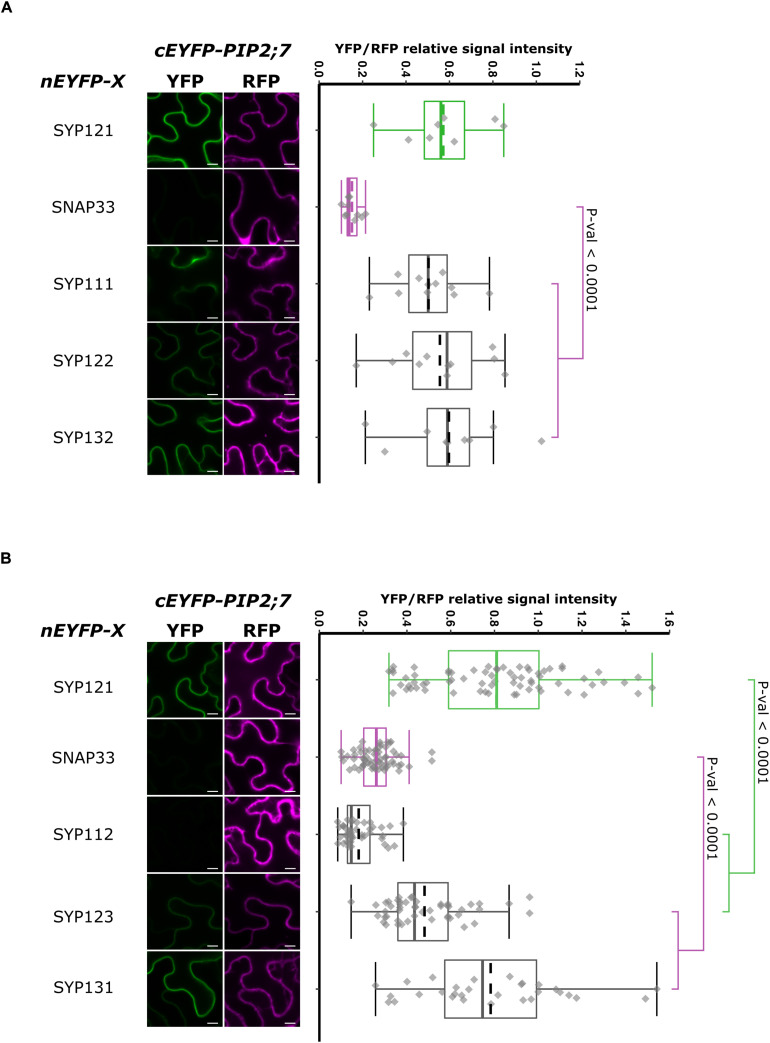
rBiFC assays for the interaction between nEYFP-SNAREs and cEYFP-PIP2;7 pairs. **(A)** rBiFC assays for the interaction between nEYFP-SYP1s and cEYFP-PIP2;7. **On the left:** representative rBiFC images. Images on the left show the YFP signal resulting from protein interaction, while those on the right show the control RFP signal. The scale bar represents 10 μm. **On the right:** ratiometric quantification of the fluorescent signals. Between 19 and 22 cells for each protein pair were analyzed by pair as described in Figure 1. **(B)** rBiFC assays for the interaction between nEYFP-SYP1s and cEYFP-PIP2;7. **On the left:** representative rBiFC images. Images on the left show the YFP signal resulting from protein interaction, while those on the right show the control RFP signal assessing the expression level after leaf infiltration. The scale bar represents 10 μm. **On the right:** ratiometric quantification of the fluorescent signals. Between 30 and 49 cells for each protein pair were analyzed by pair as described in Figure 1.

In order to have a more complete picture of the syntaxins that interact with PIP2;7, we screened the interaction of the latter with the other members of the Arabidopsis SYP1 subfamily, which are the closest homologs of SYP121 (Uemura et al., 2004; Bassham et al., 2008). The rBiFC assays revealed that only SYP112/PIP2;7 pair did not lead to the production of a fluorescent rBiFC signal, suggesting that these proteins did not physically interact (Figure 8 and Supplementary Figure 5). A fluorescent signal was detected when PIP2;7 was expressed with the other SYP1s indicating an interaction. The YFP/RFP fluorescence signal ratios confirmed the rBiFC visual observations. The rBiFC ratio for SYP123/PIP2;7 was different from both positive and negative controls (Figure 8B).

**FIGURE 9:**
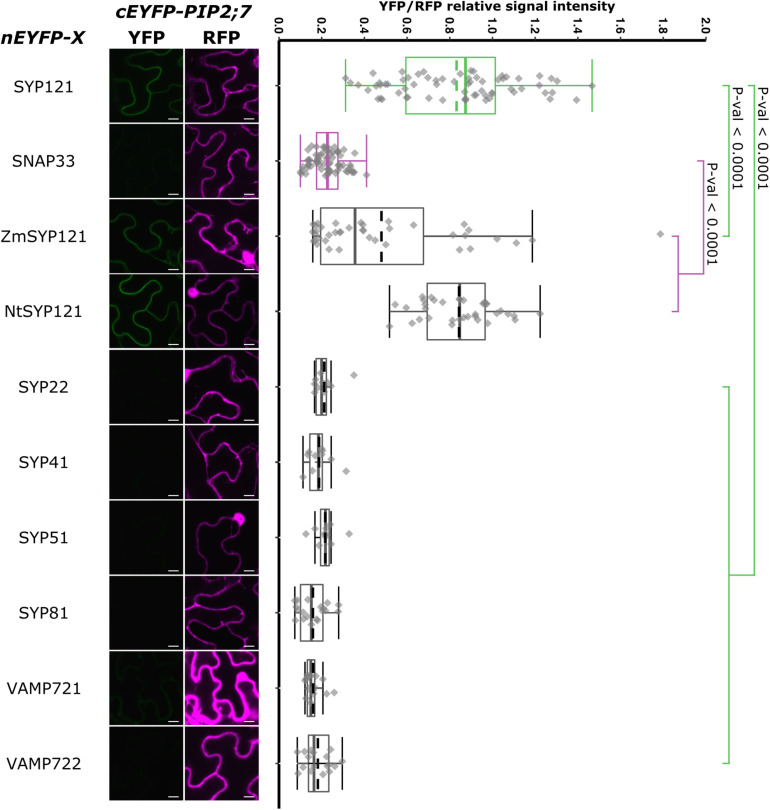
rBiFC assays for the interaction between nEYFP-SYP121 orthologs or nEYFP-SNAREs and cEYFP-PIP2;7. **On the left:** representative rBiFC images. The left images show the YFP signal resulting from protein interaction, while those on the right show the control RFP signal. The scale bar represents 10 μm. **On the right:** ratiometric quantification of the fluorescent signals. Between 10 and 40 cells for each protein pair were analyzed by pair as described in Figure 1.

We also analyzed the putative interaction between PIP2;7 and (i) SYP121 orthologs to verify whether the PIP2–SYP121 interaction is conserved through evolution, and (ii) SNAREs localized in other compartments than the plasma membrane, but transiting to the secretory pathway as PIP2;7. Maize and tobacco SYP121 share, respectively, 60% and 72% sequence identity with AtSYP121. NtSYP121 was selected to test its interaction with PIP2;7 as the work done on SYP121 and K^+^ channels originates from the characterization of NtSYP121 and its impact on K^+^ and Cl^–^ channels in stomatal guard cells (Leyman et al., 1999, 2000; Geelen et al., 2002). ZmSYP121 was the first syntaxin shown to physically interact with an aquaporin, ZmPIP2;5 (Besserer et al., 2012). SYP22, SYP41, and SYP81 are Qa-SNARE localized at the vacuole, TGN and ER, respectively (Uemura et al., 2004). SYP51 is a Qc-SNARE localized at the vacuole that was recently shown to interact with NIP1;1 (Uemura et al., 2004; Barozzi et al., 2019). Ratiometric BiFC experiments were performed and a YFP signal was observed for NtSYP121/PIP2;7 and ZmSYP121/PIP2;7 but not the other SNARE/PIP2;7 pairs (Figure 9). Quantification of the fluorescent ratios confirmed that the ratio for ZmSYP121/PIP2;7 was significantly different from both the positive and negative controls. We also tested a potential interaction between PIP2;7 and VAMP721 or VAMP722, members of the SYP121 SNARE complex together with SNAP33 (Kwaaitaal et al., 2010). No fluorescent signal was detected, indicating that these VAMP proteins did not physically interact with PIP2;7 (Figure 9). VAMP721 was recently shown to physically interact with shakers K^+^ channels to regulate their activity in the plasma membrane (Zhang et al., 2015, 2017). Our data indicated that this regulation did not take place with PIP2;7.

## Discussion

SYP121 physically interacts with PIP aquaporins to regulate their trafficking and activity (Besserer et al., 2012; Hachez et al., 2014). Identifying the interaction motifs in both proteins would allow to design new experiments to better understand the physiological role of this protein interaction. We based our search for these motifs on quantitative ratiometric BiFC assays (Grefen and Blatt, 2012) using transiently expressed proteins in *N. benthamiana* and systematically used positive and negative controls to validate the data. The interaction between some modified/mutated/truncated versions of the SYP121 and PIP2;7 proteins will have to be validated using another approach and ideally in stable Arabidopsis transformants using co-immunoprecipitation, as demonstrated with intact SYP121 and PIP2;7 proteins (Hachez et al., 2014). These lines will also be very useful to investigate the physiological relevance of this physical interaction.

### SYP121 Motifs

We first demonstrated that the FxRF motif involved in the interaction with KC1 was not required for SYP121/PIP2;7 (Figure 1), making the initial hypothesis of a PIP/SYP121/K^+^ channel tripartite still valid, as different motifs in SYP121 are required for its interaction with both PIP2;7 and KC1. It is well known that proteins can carry several interaction motifs, allowing interactions with distinct proteins at the same time to form complexes but also to compete if a motif is shared (Van Roey et al., 2014). Such a PIP/SYP121/K^+^ channel tripartite regulation could coordinate the turgor sensing with a tight tuning of ions and water movement and content in growing cells, guard cells, or cells submitted to drought stress (Besserer et al., 2012; Hachez et al., 2014).

Analysis of SYP121 deletions showed that the HQ and C regions were both interacting with PIP2;7 (Figure 1). Indeed, the C-terminus was sufficient to detect an interaction, while shortening the protein from the C- and N-terminal ends indicated that SYP121-HQ, but not SYP121ΔQC, SYP121-H, and SYP121-Q were still able to interact with PIP2;7 (Figure 2). In addition, the region between the H and Q domains and more precisely the amino acid residues 171–222, were involved in the interaction. We therefore propose the presence of two separate interaction motifs in SYP121, as previously reported in Sed5, a *Saccharomyces cerevisiae* SNARE, for its interaction with Sec23/24 (Mossessova et al., 2003). Two distinct motifs in VAMP721 have also been shown to be involved in its interaction with KAT1 (Zhang et al., 2017).

The interaction observed for the pair SYP121ΔNHQ/PIP2;7 (Figure 1) might be surprising as the deletion only contains a TM as well as a ∼40 residue long extracellular C-terminus. Such interaction was also observed in rBiFC assays between SYP121ΔNHQ and SNAP33 using similar conditions (Hecker et al., 2015), but this interaction was unexpected because the SYP121 Qa-SNARE domain, supposed to be involved in the interaction with SNAP33, was absent in the deletion. In addition, this interaction was not confirmed in Förster Resonance Energy Transfer (FRET). Therefore, the authors suggested that this could be due to the potential of BiFC split fluorophore reconstitution to enhance weak and transient interactions (Hecker et al., 2015). In addition, as Qa-SNAREs are able to assemble in homomultimer and heterodimer with Qb+Qc SNAREs, the single TM domain could interact with the *N. benthamiana* SYP121 homolog in a complex with SNAP33 and therefore bringing in close proximity the half EYFP for reconstitution, explaining the observed interaction in rBiFC (Hecker et al., 2015). The interaction observed for SYP121ΔNHQ/PIP2;7 pair could also be explained by the interaction of SYP121ΔNHQ with NbSYP121, which interacts with PIP2;7. PIP2;7 interaction with NbSYP121 should be confirmed but as its close ortholog NtSYP121 was interacting (Figure 9), an interaction can be expected. On the other hand, our laboratory identified an ER export motif in the TM3 of ZmPIP2;5, highlighting that trafficking motifs can be embedded within membrane, and putatively interact with another membrane protein (Chevalier et al., 2014). Therefore, it is possible that the PIP2;7 interaction motif in SYP121ΔNHQ was localized within the SYP121 TM. The ∼40 amino acid long extracellular sequence could alternatively be the region in which the interaction occurs. In this case, it would interact with PIP sequence facing the extracellular face. The PIP2;7 extracellular loop C, which was shown to be required for PIP2;7 interaction with SYP121 (Figure 7), might be a possible target.

Regarding the interaction motif in the HQ region, neither the H nor the Q region interacted alone with PIP2;7 while the residues [171–222], including the linker as well the N-terminus of the Qa-SNARE domain, led to a weak interaction (Figure 2). This suggested that other parts of the HQ region were required for the interaction and that the motif could be dependent on the conformation taken by SYP121. Indeed, Qa-SNAREs switch from a closed conformation, in which the three helixes of the Habc domain fold back on the SNARE domain, bringing in close proximity the H and Q regions and preventing the SNARE complex formation, to an open conformation, in which the Qa-SNARE domain is free to interact with other SNAREs for membrane fusion (Sutton et al., 1998; Jahn and Scheller, 2006; Grefen and Blatt, 2008). One hypothesis is that SYP121-HQ interacts with PIP2;7 in closed conformation, explaining why both the H and Q regions are required, with the involvement of the linker that, without a proper conformation, cannot interact sufficiently. How this putative interaction with the closed conformation of SYP121 participates in or interferes with the function of the latter will have to be investigated. An effect of the conformational state, hiding or not the motif, was observed for the VAMP721 interaction with K^+^ channels (Zhang et al., 2017). Point mutations in the linker between the Habc and Qa-SNARE domains are known to allow the stabilization of the open conformation (Dulubova et al., 1999). In Karnik et al. (2013), two-point mutations, L185A and D186A, were used to generate a stabilized open conformation of SYP121. The same mutation could be inserted in SYP121-HQ as well as WT SYP121 to investigate whether these mutated forms still interact with PIP2;7.

In a recent paper reporting the interaction between NIP1;1 and SYP51, Barozzi et al. (2019), identified H3, the Qc-SNARE domain and the TM of SYP51 (corresponding to the SYP121ΔNH construct), as the region involved in the interaction with NIP1;1. As they did not analyze other SYP51 regions, we cannot rule out that other domains were also involved in the interaction as for SYP121/PIP2;7 interaction.

### PIP2;7 Motifs

Deletion for both the N- and/or C-termini of PIP2;7 abolished the interaction with SYP121, suggesting that both termini are required (Figure 3). These PIP2;7 deleted forms were still partly localized in the plasma membrane (Supplementary Figure 2B). Using shorter N-terminus deletions, we identified the amino acid residues located between Y17 and D27 as necessary for the interaction (Figure 4). On the other hand, all the shorter deletions in the C-terminus led to weaker interaction signals but none of them totally abolished them (Figure 5). Therefore, the C-terminus might be important for the proper folding of the N-terminus rather than being directly involved in the interaction with SYP121. Indeed, looking at the structure of SoPIP2;1 (Tornroth-Horsefield et al., 2006; Nyblom et al., 2009), which shares 86% of sequence identity with PIP2;7, most of the N- and C-termini appear highly flexible and contribute the pore gating, interacting with the loop D. The N-terminus interacts with the loops B and D, through the Asp28, Gly30, and Glu31 and the binding of divalent cations, to stabilize the closed conformation by anchoring the loop D, which occludes the pore. In the open conformation, these interactions are disrupted. The PIP2;7 C-terminus also interacts with the loop D, but of an adjacent monomer in the tetramer to promote the closed conformation (Tornroth-Horsefield et al., 2006; Nyblom et al., 2009). Upon phosphorylation of the Ser274 and Ser273, the whole C-terminus was disordered, releasing the loop D and promoting the pore opening. In the SYP121/PIP2;7 interaction study, different YFP/RFP ratios were obtained for the pair SYP121/PIP2;7ΔCt and the pairs SYP121/PIP2;7–266Δ, SYP121/PIP2;7–270Δ, and SYP121/PIP2;7–274Δ (Figure 5), indicating that several residues are involved in this interaction. In the N-terminus, the three amino acid residues involved in the loop D anchoring are strictly conserved in PIP2;7, albeit at position 27, 29, and 30. As no interaction with SYP121 was observed for SYP121/PIP2;7Δ1–26 (Figure 4), the interaction motif seems not to include these three residues. Whether the binding of SYP121 to the PIP2;7 N-terminus influence the channel gating will have to be determined.

The swapping experiments between PIP2;7 and PIP2;6, a PIP isoform that does not interact with SYP121, indicated that, in addition to the role of PIP2;7 N-terminus in the interaction, the latter was not sufficient and required also a N-terminal region up to the loop C to fully restore the interaction signal. However, it would be necessary to confirm that the PIP2;7][PIP2;6 complementary swappings of the loops and TMs (PIP2;6-B][TM3-PIP2;7, PIP2;6-TM3][C-PIP2;7, PIP2;6-C][TM4-PIP2;7, and PIP2;6-D][TM5-PIP2;7) do not interact. Altogether, the data from both the swapping and deletions experiments indicate that the interaction between PIP2;7 and SYP121 requires a region located between the residue 18 and the loop C of PIP2;7, with a crucial role of the residues 18–26, and also a contribution of the C-terminus. This could be confirmed by the complementary set of PIP2;6][PIP2;7 TM and loop swapped isoforms. Similar to SYP121-HQ, a conformational motif depending on the complex array of interaction between the N-and C-termini and the N-terminal loops could be essential for the interaction with SYP121, explaining why removing either the N- or C-terminus impaired the interaction. On the other hand, it is not clear why the extracellular loop C was required. It could interact with the C-terminus of SYP121 or be involved in maintaining the proper protein conformation for the interaction.

### SYP Interaction With PIP2;7

All the Arabidopsis SYP1s except SYP112 interact with PIP2;7 (Figure 8 and Supplementary Figure 5). Comparison of the expression pattern of each of them (Enami et al., 2009) with the one of PIP2;7 suggest that such interactions might take places *in planta*. PIP2;7 is expressed in the rosette, flowers and roots, excluding the root tips (Hachez et al., 2014). In the leaf, it is expressed in the veins and the mesophyll cells but more highly in the primordia. Similar expression patterns are found for SYPs (Enami et al., 2009). SYP111 is involved in plant cytokinesis and is thus specifically expressed in plant dividing cells, such as the leaf primordia (Enami et al., 2009), where PIP2;7 is also strongly expressed. SYP122 and SYP132 are expressed in similar tissues as PIP2;7. An interaction of SYP132 with PIP2;7 was reported in pull-down assays (Fujiwara et al., 2014). SYP123 is mostly expressed in root hairs where PIP2;7 is not expressed, but also in the rosette leaves where PIP2;7 is expressed. SYP124, SYP125 and SYP131 are specifically expressed in pollen and pollen tube (Enami et al., 2009). No interaction between SYP131 and PIP2;7 was reported in SUS assays by Jones et al. (2014) and no PIP2;7 expression was reported in the pollen in proPIP2;7:GUS lines (Da Ines, 2008; Prado et al., 2013; Hachez et al., 2014). However, large-scale data revealed that PIP2;7/2;8 (the probe used cannot differentiate between both isoforms), are expressed during pollen development but not in mature pollen (Pérez Di Giorgio et al., 2016).

In Hachez et al. (2014), co-expression of SYP121ΔC and EYFP-PIP2;7 impaired the trafficking of the later and reduced the P_f_ of EYFP-PIP2;7 expressing protoplasts. Therefore, it would be interesting to co-express each SYP1ΔC fragment together with EYFP-PIP2;7 in protoplasts to study the impact of individual SYP1 isoforms on the PIP2;7 trafficking and activity. In maize, ZmPIP2;5 trafficking was impaired by ZmSYP121ΔC but not by AtSYP122ΔC, AtSYP21ΔC, and AtSYP71ΔC (Besserer et al., 2012). However, in that case, no physical interactions between these SYP proteins and ZmPIP2;5 were assessed. PIP2;7 function in plants was only linked to water movement so far (Weig et al., 1997; Jang et al., 2004; Alexandersson et al., 2005; Prado et al., 2013; Hachez et al., 2014; Pou et al., 2016). Therefore, we can assume that regulation through interactions with SYP1s takes place in this frame. On the other hand, the study of SYP1s/PIP2;7 interactions could highlight new roles for PIP2;7.

Other plasma membrane localized SNAREs were tested for their interactions with PIP2;7 (Figure 9). The other members of the SYP121 cognate SNARE complex, VAMP721/722 and SNAP33, did not interact with PIP2;7 in rBiFC assays. The absence of interaction between SNAP33 and PIP2;7 was used as a negative control throughout this study. In that case, SNAP33 and VAMP721 were already expressed in other studies using the same pBiFCt-2in1 vectors and we can thus rule out the absence of expression to explain the absence of interaction (Hecker et al., 2015; Zhang et al., 2015, 2017; Xing et al., 2016). SNAREs with other subcellular localizations such as SYP22, SYP41, SYP51, and SYP81 did not interact with PIP2;7 in rBiFC assays. According to RT-PCR data, these four SNAREs are expressed in the flowers, leaves, stems, and roots (Uemura et al., 2004). This absence of interaction is consistent with the results obtained in Tyrrell et al. (2007) showing that the vacuolar TIP1;1 trafficking is impaired by the PVC/vacuolar localized SYP21ΔC but not SYP121ΔC. PIP2;7 interacts with the TGN localized SYP61 to regulates its trafficking to the plasma membrane (Hachez et al., 2014). SYP61 is known to form SNARE complexes with the TGN-localized SYP41 (Drakakaki et al., 2012), which does not interact with PIP2;7. Therefore, it seems that plant aquaporins, specifically interact with SNAREs involved in the trafficking to their resident membranes.

### Physiological Meaning of PIP/SYP121 Interaction

Most PIPs interact with SYP121, indicating a conserved mechanism of PIP regulation by SYP121 (Supplementary Figure 4; Besserer et al., 2012; Hachez et al., 2014). Considering that plant must fine-tune their water content for optimal growth and development, such fast general mechanism would make sense in order to regulate the water movement at the plasma membrane, in close coordination with the trafficking and activity of K^+^ channels, key actors in cell water osmoregulation. Consistent with this, the constitutive expression of the SYP121ΔC fragment impairs growth and development of tobacco plants (Geelen et al., 2002; Tyrrell et al., 2007). To confirm that a general regulation mechanism takes place, co-expression of SYP121ΔC together with an EYFP-PIP versions in protoplasts would allow assessing the impact of SYP121ΔC on the trafficking of each PIPs isoform *en route* to the plasma membrane. It could also be possible that the interaction regulates the activity of PIPs at the plasma membrane in addition to their trafficking. Such impact was observed in Besserer et al. (2012). Indeed, upon co-expression of ZmSYP121ΔC, but not WT ZmSYP121, with ZmPIP2;5 in oocytes, a P_f_ reduction was observed that was not linked to a reduction in ZmPIP2;5 abundance in the plasma membrane. As mentioned above, a regulation of SYP121 by PIPs is also possible but would need further investigation.

Arabidopsis PIPs are also permeable to other solutes than water such as H_2_O_2_ (Dynowski et al., 2008; Hooijmaijers et al., 2012; Tian et al., 2016; Rodrigues et al., 2017). Looking whether the interaction impacts such transport could highlight new SYP121 functions. For instance, SYP121 is known to be involved in the resistance to powdery mildew infection (Collins et al., 2003; Assaad et al., 2004; Kwon et al., 2008; Pajonk et al., 2008). Upon infection, SYP121 accumulates to the papilla in which an H_2_O_2_ accumulation was also observed (Király et al., 2007). Regulating the transport of H_2_O_2_ by PIPs could prevent this infection. At the guard cells of stomatal complexes, NtSYP121 is involved in the K^+^, Cl^–^, and Ca^++^ channel activity response to ABA (Leyman et al., 1999; Sokolovski et al., 2008). The involvement of AtSYP121 in PIPs regulation in guard cells would make sense to regulate both the water and H_2_O_2_ membrane diffusion. PIP2;1 was reported to be involved in H_2_O_2_ and water transport in these cells (Rodrigues et al., 2017). In addition, both the PIP2;1 (Grondin et al., 2015) and NtSYP121 (Leyman et al., 1999) are involved in the ABA regulation of the stomatal closure.

## Data Availability Statement

The original contributions presented in the study are included in the article/Supplementary Material, further inquiries can be directed to the corresponding author.

## Author Contributions

TL, CH, and FC designed the experiments. TL, IM, and SB performed the experiments. TL, IM, CH, and FC analyzed the data. TL and FC wrote the manuscript. All authors contributed to the article and approved the submitted version.

## Conflict of Interest

The authors declare that the research was conducted in the absence of any commercial or financial relationships that could be construed as a potential conflict of interest.

## References

[B1] Alexandersson E. Fraysse L. Sjovall-Larsen S. Gustavsson S. Fellert M. Karlsson M. (2005). Whole gene family expression and drought stress regulation of aquaporins. *Plant Mol. Biol.* 59 469–484. 10.1007/s11103-005-0352-1 16235111

[B2] Assaad F. F. Qiu J. L. Youngs H. Ehrhardt D. Zimmerli L. Kalde M. (2004). The PEN1 syntaxin defines a novel cellular compartment upon fungal attack and is required for the timely assembly of papillae. *Mol. Biol. Cell* 15 5118–5129. 10.1091/mbc.e04-02-0140 15342780 PMC524786

[B3] Barozzi F. Papadia P. Stefano G. Renna L. Brandizzi F. Migoni D. (2019). Variation in membrane trafficking linked to SNARE AtSYP51 interaction with aquaporin NIP1;1. *Front. Plant Sci.* 9:1949. 10.3389/fpls.2018.01949 30687352 PMC6334215

[B4] Bassham D. C. Brandizzi F. Otegui M. S. Sanderfoot A. A. (2008). The secretory system of *Arabidopsis*. *Arabidopsis Book* 6:e0116. 10.1199/tab.0116 22303241 PMC3243370

[B5] Besserer A. Burnotte E. Bienert G. P. Chevalier A. S. Errachid A. Grefen C. (2012). Selective regulation of maize plasma membrane aquaporin trafficking and activity by the SNARE SYP121. *Plant Cell* 24 3463–3481. 10.1105/tpc.112.101758 22942383 PMC3462644

[B6] Chaumont F. Tyerman S. D. (2014). Aquaporins: highly regulated channels controlling plant water relations. *Plant Physiol.* 164 1600–1618. 10.1104/pp.113.233791 24449709 PMC3982727

[B7] Chevalier A. S. Bienert G. P. Chaumont F. (2014). A new LxxxA motif in the transmembrane Helix3 of maize aquaporins belonging to the plasma membrane Intrinsic protein PIP2 group is required for their trafficking to the plasma membrane. *Plant Physiol.* 166 125–138. 10.1104/pp.114.240945 24989232 PMC4149701

[B8] Collins N. C. Thordal-Christensen H. Lipka V. Bau S. Kombrink E. Qiu J. L. (2003). SNARE-protein-mediated disease resistance at the plant cell wall. *Nature* 425 973–977. 10.1038/nature02076 14586469

[B9] Da Ines O. (2008). *Functional Analysis of PIP2 Aquaporins in Arabidopsis thaliana.* Doctoral dissertation, Faculty of Chemistry and Pharmacy, LMU Munich.

[B10] Drakakaki G. Van De Ven W. Pan S. Miao Y. Wang J. Keinath N. F. (2012). Isolation and proteomic analysis of the SYP61 compartment reveal its role in exocytic trafficking in *Arabidopsis*. *Cell Res.* 22 413–424. 10.1038/cr.2011.129 21826108 PMC3271593

[B11] Dulubova I. Sugita S. Hill S. Hosaka M. Fernandez I. Sudhof T. C. (1999). A conformational switch in syntaxin during exocytosis: role of munc18. *EMBO J.* 18 4372–4382. 10.1093/emboj/18.16.4372 10449403 PMC1171512

[B12] Dynowski M. Schaaf G. Loque D. Moran O. Ludewig U. (2008). Plant plasma membrane water channels conduct the signalling molecule H_2_O_2_. *Biochem. J.* 414 53–61. 10.1042/bj20080287 18462192

[B13] Enami K. Ichikawa M. Uemura T. Kutsuna N. Hasezawa S. Nakagawa T. (2009). Differential expression control and polarized distribution of plasma membrane-resident SYP1 SNAREs in *Arabidopsis thaliana*. *Plant Cell Physiol.* 50 280–289. 10.1093/pcp/pcn197 19098073

[B14] Fasshauer D. Sutton R. B. Brunger A. T. Jahn R. (1998). Conserved structural features of the synaptic fusion complex: SNARE proteins reclassified as Q- and R-SNAREs. *Proc. Natl. Acad. Sci. U.S.A.* 95 15781–15786. 10.1073/pnas.95.26.15781 9861047 PMC28121

[B15] Fetter K. Van Wilder V. Moshelion M. Chaumont F. (2004). Interactions between plasma membrane aquaporins modulate their water channel activity. *Plant Cell* 16 215–228. 10.1105/tpc.017194 14671024 PMC301406

[B16] Fujiwara M. Uemura T. Ebine K. Nishimori Y. Ueda T. Nakano A. (2014). Interactomics of Qa-SNARE in *Arabidopsis thaliana*. *Plant Cell Physiol.* 55 781–789. 10.1093/pcp/pcu038 24556609

[B17] Geelen D. Leyman B. Batoko H. Di Sansebastiano G. P. Moore I. Blatt M. R. (2002). The abscisic acid-related SNARE homolog NtSyr1 contributes to secretion and growth: evidence from competition with its cytosolic domain. *Plant Cell* 14 387–406. 10.1105/tpc.010328 11884682 PMC152920

[B18] Grefen C. Blatt M. R. (2008). SNAREs–molecular governors in signalling and development. *Curr. Opin. Plant Biol.* 11 600–609. 10.1016/j.pbi.2008.08.006 18945636

[B19] Grefen C. Blatt M. R. (2012). A 2in1 cloning system enables ratiometric bimolecular fluorescence complementation (rBiFC). *Biotechniques* 53 311–314. 10.2144/000113941 23066669

[B20] Grefen C. Chen Z. Honsbein A. Donald N. Hills A. Blatt M. R. (2010). A novel motif essential for SNARE interaction with the K^+^ channel KC1 and channel gating in *Arabidopsis*. *Plant Cell* 22 3076–3092. 10.1105/tpc.110.077768 20884800 PMC2965544

[B21] Grefen C. Karnik R. Larson E. Lefoulon C. Wang Y. Waghmare S. (2015). A vesicle-trafficking protein commandeers Kv channel voltage sensors for voltage-dependent secretion. *Nature Plants* 1:15108. 10.1038/nplants.2015.108 27250541

[B22] Grondin A. Rodrigues O. Verdoucq L. Merlot S. Leonhardt N. Maurel C. (2015). Aquaporins contribute to ABA-triggered stomatal closure through OST1-mediated phosphorylation. *Plant Cell* 27 1945–1954. 10.1105/tpc.15.00421 26163575 PMC4531361

[B23] Hachez C. Laloux T. Reinhardt H. Cavez D. Degand H. Grefen C. (2014). *Arabidopsis* SNAREs SYP61 and SYP121 coordinate the trafficking of plasma membrane aquaporin PIP2;7 to modulate the cell membrane water permeability. *Plant Cell* 26 3132–3147. 10.1105/tpc.114.127159 25082856 PMC4145137

[B24] Hecker A. Wallmeroth N. Peter S. Blatt M. R. Harter K. Grefen C. (2015). Binary 2in1 vectors improve in planta (Co)localization and dynamic protein interaction studies. *Plant Physiol.* 168 776–787. 10.1104/pp.15.00533 25971551 PMC4741326

[B25] Honsbein A. Sokolovski S. Grefen C. Campanoni P. Pratelli R. Paneque M. (2009). A tripartite SNARE-K^+^ channel complex mediates in channel-dependent K+ nutrition in *Arabidopsis*. *Plant Cell* 21 2859–2877. 10.1105/tpc.109.066118 19794113 PMC2768940

[B26] Hooijmaijers C. Rhee J. Y. Kwak K. J. Chung G. C. Horie T. Katsuhara M. (2012). Hydrogen peroxide permeability of plasma membrane aquaporins of *Arabidopsis thaliana*. *J. Plant Res.* 125 147–153. 10.1007/s10265-011-0413-2 21390558

[B27] Jahn R. Scheller R. H. (2006). SNAREs–engines for membrane fusion. *Nature Rev. Mol. Cell Biol.* 7 631–643. 10.1038/nrm2002 16912714

[B28] Jang J. Y. Kim D. G. Kim Y. O. Kim J. S. Kang H. (2004). An expression analysis of a gene family encoding plasma membrane aquaporins in response to abiotic stresses in *Arabidopsis thaliana*. *Plant Mol. Biol.* 54 713–725. 10.1023/b:plan.0000040900.61345.a6 15356390

[B29] Jones A. M. Xuan Y. Xu M. Wang R. S. Ho C. H. Lalonde S. (2014). Border control–a membrane-linked interactome of *Arabidopsis*. *Science* 344 711–716. 10.1126/science.1251358 24833385

[B30] Kargul J. Gansel X. Tyrrell M. Sticher L. Blatt M. R. (2001). Protein-binding partners of the tobacco syntaxin NtSyr1. *FEBS Lett.* 508 253–258. 10.1016/s0014-5793(01)03089-7 11718726

[B31] Karnik R. Grefen C. Bayne R. Honsbein A. Kohler T. Kioumourtzoglou D. (2013). *Arabidopsis* Sec1/Munc18 protein SEC11 is a competitive and dynamic modulator of SNARE binding and SYP121-dependent vesicle traffic. *Plant Cell* 25 1368–1382. 10.1105/tpc.112.108506 23572542 PMC3663274

[B32] Karnik R. Waghmare S. Zhang B. Larson E. Lefoulon C. Gonzalez W. (2017). Commandeering channel voltage sensors for secretion, cell turgor, and volume control. *Trends Plant Sci.* 22 81–95. 10.1016/j.tplants.2016.10.006 27818003 PMC5224186

[B33] Kim S.-J. Brandizzi F. (2012). News and views into the SNARE complexity in *Arabidopsis*. *Front. Plant Sci.* 3:28. 10.3389/fpls.2012.00028 23018380 PMC3355637

[B34] Király L. Barna B. Király Z. (2007). Plant resistance to pathogen infection: forms and mechanisms of innate and acquired resistance. *J. Phytopathol.* 155 385–396. 10.1111/j.1439-0434.2007.01264.x

[B35] Kwaaitaal M. Keinath N. F. Pajonk S. Biskup C. Panstruga R. (2010). Combined bimolecular fluorescence complementation and Forster resonance energy transfer reveals ternary SNARE complex formation in living plant cells. *Plant Physiol.* 152 1135–1147. 10.1104/pp.109.151142 20071602 PMC2832253

[B36] Kwon C. Neu C. Pajonk S. Yun H. S. Lipka U. Humphry M. (2008). Co-option of a default secretory pathway for plant immune responses. *Nature* 451 835–840. 10.1038/nature06545 18273019

[B37] Lazo G. R. Stein P. A. Ludwig R. A. (1991). A DNA transformation-competent *Arabidopsis* genomic library in *Agrobacterium*. *Biotechnology (N Y)* 9 963–967. 10.1038/nbt1091-963 1368724

[B38] Lefoulon C. Waghmare S. Karnik R. Blatt M. R. (2018). Gating control and K^+^ uptake by the KAT1 K^+^ channel leaveraged through membrane anchoring of the trafficking protein SYP121. *Plant Cell Environ.* 41 2668–2677. 10.1111/pce.13392 29940699 PMC6220998

[B39] Leyman B. Geelen D. Blatt M. R. (2000). Localization and control of expression of Nt-Syr1, a tobacco SNARE protein. *Plant J. Cell Mol. Biol.* 24 369–381. 10.1046/j.1365-313x.2000.00886.x 11069710

[B40] Leyman B. Geelen D. Quintero F. J. Blatt M. R. (1999). A tobacco syntaxin with a role in hormonal control of guard cell ion channels. *Science* 283 537–540. 10.1126/science.283.5401.537 9915701

[B41] Lipka V. Kwon C. Panstruga R. (2007). SNARE-ware: the role of SNARE-domain proteins in plant biology. *Annu. Rev. Cell Dev. Biol.* 23 147–174. 10.1146/annurev.cellbio.23.090506.123529 17506694

[B42] Mossessova E. Bickford L. C. Goldberg J. (2003). SNARE selectivity of the COPII coat. *Cell* 114 483–495. 10.1016/s0092-8674(03)00608-1 12941276

[B43] Nyblom M. Frick A. Wang Y. Ekvall M. Hallgren K. Hedfalk K. (2009). Structural and functional analysis of SoPIP2;1 mutants adds insight into plant aquaporin gating. *J. Mol. Biol.* 387 653–668. 10.1016/j.jmb.2009.01.065 19302796

[B44] Pajonk S. Kwon C. Clemens N. Panstruga R. Schulze-Lefert P. (2008). Activity determinants and functional specialization of *Arabidopsis* PEN1 syntaxin in innate immunity. *J. Biol. Chem.* 283 26974–26984. 10.1074/jbc.m805236200 18678865

[B45] Pérez Di Giorgio J. A. Soto G. C. Muschietti J. P. Amodeo G. (2016). Pollen aquaporins: the solute factor. *Front. Plant Sci.* 7:1659. 10.3389/fpls.2016.01659 27881985 PMC5101680

[B46] Pou A. Jeanguenin L. Milhiet T. Batoko H. Chaumont F. Hachez C. (2016). Salinity-mediated transcriptional and post-translational regulation of the *Arabidopsis* aquaporin PIP2;7. *Plant Mol. Biol.* 92 731–744. 10.1007/s11103-016-0542-z 27671160

[B47] Prado K. Boursiac Y. Tournaire-Roux C. Monneuse J. M. Postaire O. Da Ines O. (2013). Regulation of *Arabidopsis* leaf hydraulics involves light-dependent phosphorylation of aquaporins in veins. *Plant Cell* 25 1029–1039. 10.1105/tpc.112.108456 23532070 PMC3634675

[B48] R Core Team (2019). *R: A Language and Environment for Statistical Computing.* Vienna: R Foundation for Statistical Computing.

[B49] Rodrigues O. Reshetnyak G. Grondin A. Saijo Y. Leonhardt N. Maurel C. (2017). Aquaporins facilitate hydrogen peroxide entry into guard cells to mediate ABA- and pathogen-triggered stomatal closure. *Proc. Natl. Acad. Sci. U.S.A.* 114 9200–9205. 10.1073/pnas.1704754114 28784763 PMC5576802

[B50] Sanderfoot A. (2007). Increases in the number of SNARE genes parallels the rise of multicellularity among the green plants. *Plant Physiol.* 144 6–17. 10.1104/pp.106.092973 17369437 PMC1913785

[B51] SAS Institute Inc (2016). *SAS Entreprise Guide (V7.13).* Cary, NC: SAS Institute Inc.

[B52] Sokolovski S. Hills A. Gay R. A. Blatt M. R. (2008). Functional interaction of the SNARE protein NtSyp121 in Ca^2+^ channel gating, Ca^2+^ transients and ABA signalling of stomatal guard cells. *Mol. Plant* 1 347–358. 10.1093/mp/ssm029 19825544

[B53] Sutter J. U. Campanoni P. Tyrrell M. Blatt M. R. (2006). Selective mobility and sensitivity to SNAREs is exhibited by the Arabidopsis KAT1 K^+^ channel at the plasma membrane. *Plant Cell* 18 935–954. 10.1105/tpc.105.038950 16531497 PMC1425843

[B54] Sutton R. B. Fasshauer D. Jahn R. Brunger A. T. (1998). Crystal structure of a SNARE complex involved in synaptic exocytosis at 2.4 Å resolution. *Nature* 395 347–353. 10.1038/26412 9759724

[B55] Tian S. Wang X. Li P. Wang H. Ji H. Xie J. (2016). Plant aquaporin AtPIP1;4 links apoplastic H_2_O_2_ induction to disease immunity pathways. *Plant Physiol.* 171 1635–1650. 10.1104/pp.15.01237 26945050 PMC4936539

[B56] Tornroth-Horsefield S. Wang Y. Hedfalk K. Johanson U. Karlsson M. Tajkhorshid E. (2006). Structural mechanism of plant aquaporin gating. *Nature* 439 688–694. 10.1038/nature04316 16340961

[B57] Tyrrell M. Campanoni P. Sutter J. U. Pratelli R. Paneque M. Sokolovski S. (2007). Selective targeting of plasma membrane and tonoplast traffic by inhibitory (dominant-negative) SNARE fragments. *Plant J. Cell Mol. Biol.* 51 1099–1115. 10.1111/j.1365-313x.2007.03206.x 17662029

[B58] Uemura T. Ueda T. Ohniwa R. L. Nakano A. Takeyasu K. Sato M. H. (2004). Systematic analysis of SNARE molecules in *Arabidopsis*: dissection of the post-Golgi network in plant cells. *Cell Struct. Funct.* 29 49–65. 10.1247/csf.29.49 15342965

[B59] Van Roey K. Uyar B. Weatheritt R. J. Dinkel H. Seiler M. Budd A. (2014). Short linear motifs: ubiquitous and functionally diverse protein interaction modules directing cell regulation. *Chem. Rev.* 114 6733–6778. 10.1021/cr400585q 24926813

[B60] Weig A. Deswarte C. Chrispeels M. J. (1997). The major intrinsic protein family of *Arabidopsis* has 23 members that form three distinct groups with functional aquaporins in each group. *Plant Physiol.* 114 1347–1357. 10.1104/pp.114.4.1347 9276952 PMC158427

[B61] Wickman H. (2009). *ggplot2: Elegant Graphics for Data Analysis.* New York, NY: Springer-Verlag.

[B62] Xing S. Wallmeroth N. Berendzen K. W. Grefen C. (2016). Techniques for the Analysis of Protein-Protein Interactions in Vivo. *Plant Physiol.* 171 727–758. 10.1104/pp.16.00470 27208310 PMC4902627

[B63] Zelazny E. Borst J. W. Muylaert M. Batoko H. Hemminga M. A. Chaumont F. (2007). FRET imaging in living maize cells reveals that plasma membrane aquaporins interact to regulate their subcellular localization. *Proc. Natl. Acad. Sci. U.S.A.* 104 12359–12364. 10.1073/pnas.0701180104 17636130 PMC1941474

[B64] Zelazny E. Miecielica U. Borst J. W. Hemminga M. A. Chaumont F. (2009). An N-terminal diacidic motif is required for the trafficking of maize aquaporins ZmPIP2;4 and ZmPIP2;5 to the plasma membrane. *Plant J.* 57 346–355. 10.1111/j.1365-313x.2008.03691.x 18808456

[B65] Zhang B. Karnik R. Waghmare S. Donald N. Blatt M. R. (2017). VAMP721 conformations unmask an extended motif for K^+^ channel binding and gating control. *Plant Physiol.* 173 536–551. 10.1104/pp.16.01549 27821719 PMC5210753

[B66] Zhang B. Karnik R. Wang Y. Wallmeroth N. Blatt M. R. Grefen C. (2015). The *Arabidopsis* R-SNARE VAMP721 interacts with KAT1 and KC1 K^+^ channels to moderate K^+^ current at the plasma membrane. *Plant Cell* 27 1697–1717. 10.1105/tpc.15.00305 26002867 PMC4498211

