# Supplementary material for: Interaction Between the SNARE SYP121 and the Plasma Membrane Aquaporin PIP2;7 Involves Different Protein Domains
Source: Front Plant Sci. 2021 Jan 18;11:631643. doi: 10.3389/fpls.2020.631643 (PMC7847993; doi:10.3389/fpls.2020.631643)
Supplement: Supplementary file 1 [file Data_Sheet_1.PDF]

Supplemental Figure 1

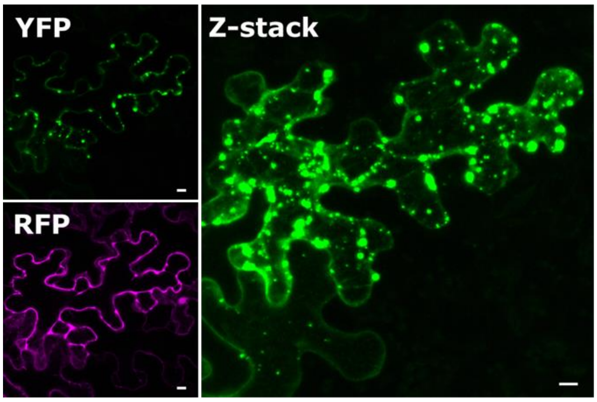

Supplemental Figure 1. cEYFP-PIP2;7/nEYFP-SYP121ΔNH interaction signals accumulated in internal structures. Top left image shows the YFP signal, resulting from protein interaction. The bottom left image shows the RFP, the internal marker of the infiltration. Image on the right is a 3D reconstitution by maximum projection of stacked confocal images. The scale bar represents 10 µm.

Supplemental Figure 2

A

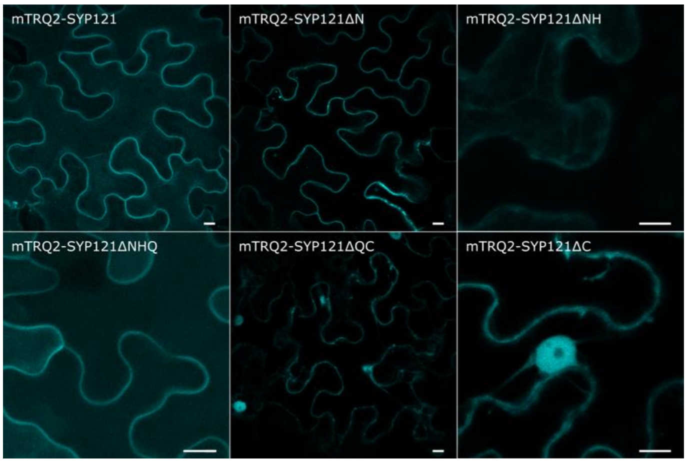

B

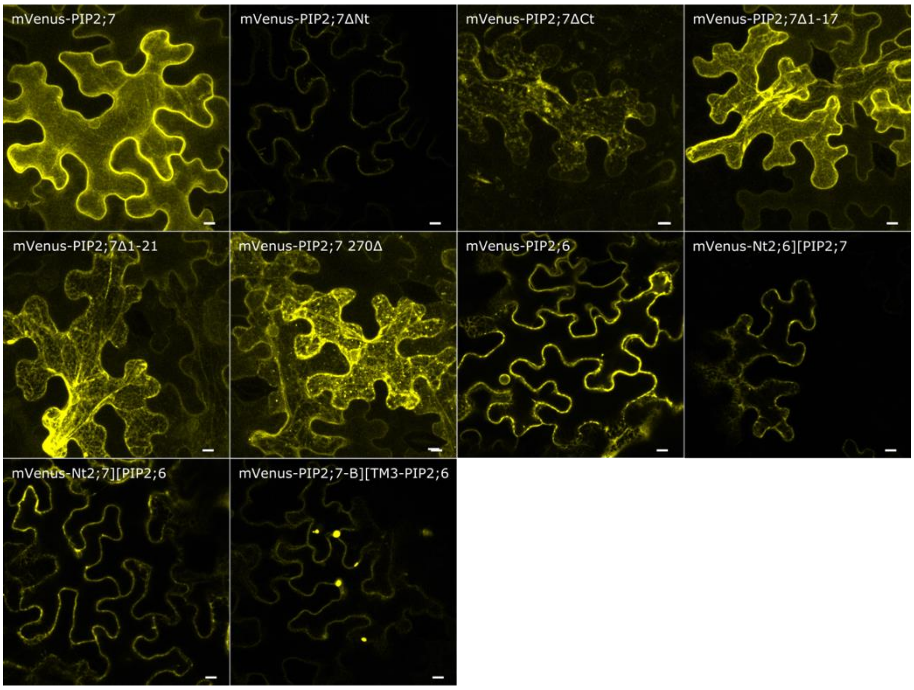

Supplemental Figure 2. Subcellular localization of different SYP121 and PIP2;7 forms fused to mTRQ2 and mVenus, respectively, and expressed in *N. benthamiana* cells. A. Confocal microscopy images of the subcellular localization of mTRQ2-SYP121 and mTRQ2-SYP121 deletions. A signal was observed in the plasma membrane for mTRQ2-SYP121, mTRQ2-SYP121ΔN, mTRQ2-SYP121ΔNH and mTRQ2-SYP121ΔNHQ. A signal was observed in the cytoplasm for mTRQ2-SYP121ΔQC and mTRQ2-SYP121ΔC. **B.** Confocal microscopy images of the subcellular localization of mVenus-PIP2;7, mVenus-PIP2;7 deletions and mVenus-PIP2;7][PIP2;6 chimeric proteins. A signal was observed in the plasma membrane and in internal structures for all tested proteins. The scale bar represents 10 µm.

Supplemental Figure 3

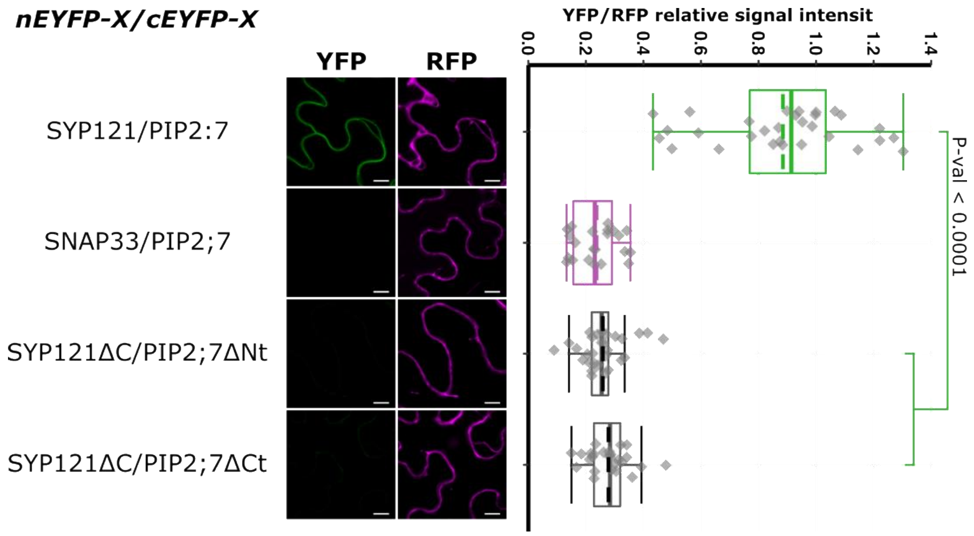

Supplemental Figure 3. rBiFC assays for cEYFP-PIP2;7 deletions and nEYFP-SYP121ΔC pairs. On the left: representative rBiFC images. Images on the left show the YFP signal resulting from protein interaction, while those on the right show the control RFP signal. The scale bar represents 10 µm. On the right: ratiometric quantification of the fluorescent signals. Between 26 and 29 cells for each protein pair were analyzed by pair as described in Figure 1.

Supplemental Figure 4

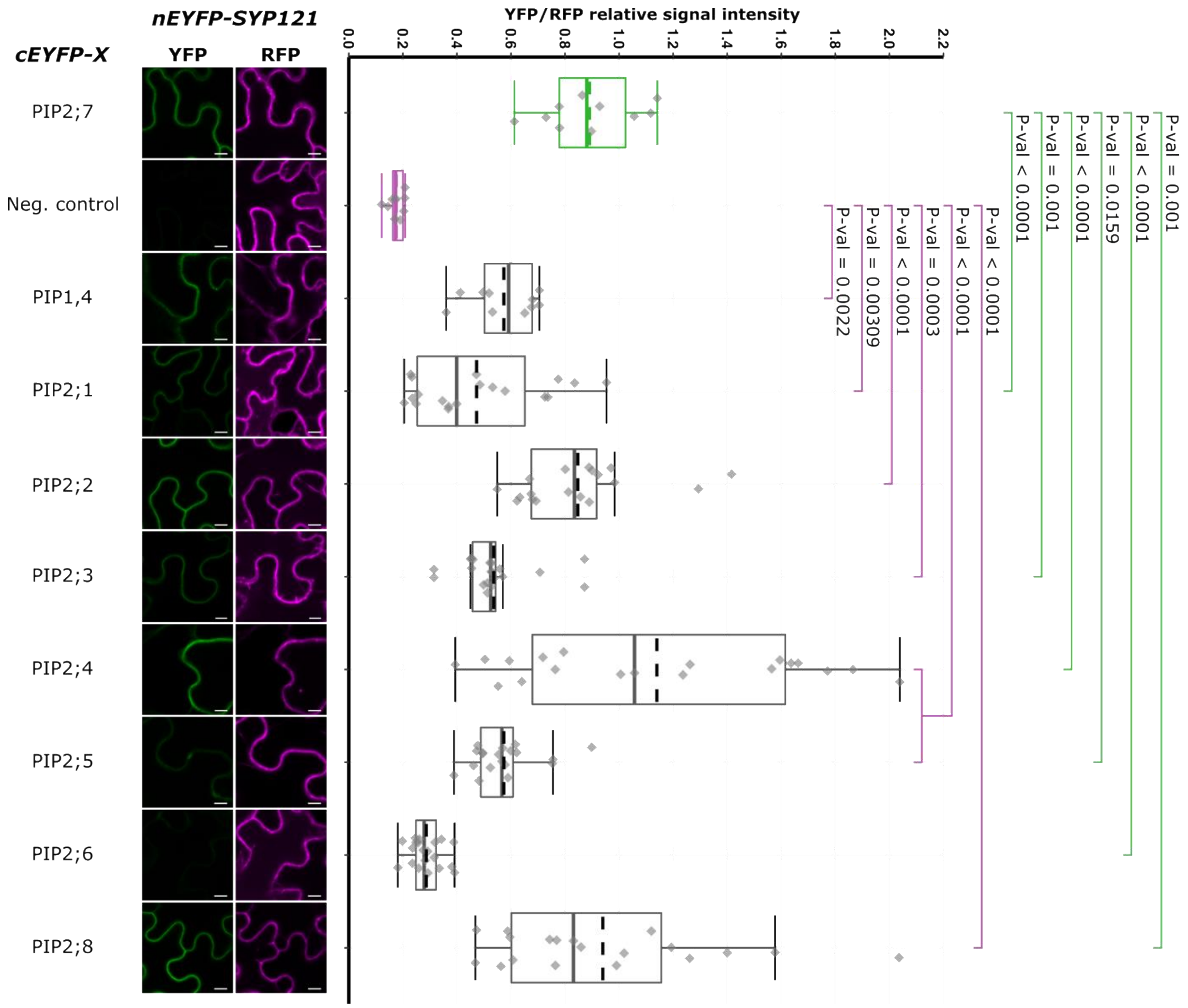

Supplemental Figure 4. BiFC assays for the interaction between cEYFP-PIPs and nEYFP-SYP121. On the left: representative rBiFC images. Images on the left show the YFP signal resulting from protein interaction, while those on the right show the control RFP signal. The scale bar represents 10 µm. On the right: ratiometric quantification of the fluorescent signals. Between 10 and 20 cells for each protein pair were analyzed by pair as described in Figure 1. The negative control is the nEYFP-SNAP33/cEYFP-PIP2;7 pair.

Supplemental Figure 5

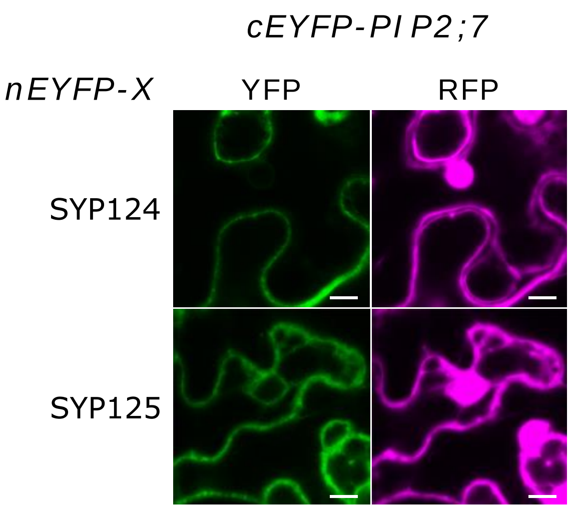

Supplemental Figure 5. rBiFC signals for nEYFP-SYP124, nEYFP-SYP125 and cEYFP-PIP2;7 pairs. SYP124 and SYP125 were tested for their interaction with PIP2;7. The left panels show the YFP signal, resulting from protein interaction. The right panels show the RFP, the internal marker of the infiltration. A YFP signal was observed for nEYFP-SYP124/cEYFP-PIP2;7 and nEYFP-SYP125/cEYFP-PIP2;7. The scale bar represents 10 µm.
