# Supplementary material for: Interaction Between the SNARE SYP121 and the Plasma Membrane Aquaporin PIP2;7 Involves Different Protein Domains
Source: Front Plant Sci. 2021 Jan 18;11:631643. doi: 10.3389/fpls.2020.631643 (PMC7847993; doi:10.3389/fpls.2020.631643)
Supplement: Supplementary file 2 [file Table_1.pdf]

## Supplemental Table 1. Primer list

| Primer | Sequence |
|---|---|
| attB3AtSYP121_Fw | 5′ – GGGG *ACA ACT TTG TAT AAT AAA GTT G*TA ATG AAC GAT TTG TTT TCC AGC – 3′ |
| attB2AtSYP121_Rv | 5′ – GGGG *AC CAC TTT GTA CAA GAA AGC TGG GT*T TCA ACG CAA TAG ACG CCT TGC – 3′ |
| attB3AtSYP121ΔN_Fw | 5′ – GGGG *ACA ACT TTG TAT AAT AAA GTT G*TA GGT GGT GTG AAC CTC GAC AAG – 3′ |
| attB3AtSYP121ΔNH_Fw | 5′ – GGGG *ACA ACT TTG TAT AAT AAA GTT G*TA GAG AGA TTC TTG CAG AAA GCA – 3′ |
| attB3AtSYP121ΔNHQ_Fw | 5′ – GGGG *ACA ACT TTG TAT AAT AAA GTT G*TA CGA AAA TGG ACA TGT ATT GCC – 3′ |
| attB2AtSYP121ΔC_Rv | 5′ – GGGG *AC CAC TTT GTA CAA GAA AGC TGG GT*T TCA TCG CGT GTT CTT CTG GTA AAC – 3′ |
| attB2AtSYP121ΔQC_Rv | 5′ – GGGG *AC CAC TTT GTA CAA GAA AGC TGG GT*T TTA TCC AGT GGA AAT CAG TCG ATC – 3′ |
| attB3AtSYP121-[143_Fw | 5′ – GGGG *ACA ACT TTG TAT AAT AAA GTT G*TA AGG AAG AAA TTG ATG GAC TCT – 3′ |
| attB2AtSYP121-222]_Rv | 5′ – GGGG *AC CAC TTT GTA CAA GAA AGC TGG GT*T TCA ATC ATG CCT TTC TTG AAT – 3′ |
| attB3AtSYP121-[171_Fw | 5′ – GGGG *ACA ACT TTG TAT AAT AAA GTT G*TA AGG TAC TTC ACC GTC ACC GGC – 3′ |
| attB2AtSYP121-200]_Rv | 5′ – GGGG *AC CAC TTT GTA CAA GAA AGC TGG GT*T TCA TTT CTG CAA GAA TCT CTC – 3′ |
| attB3AtSYP111_Fw | 5′ – GGGG *ACA ACT TTG TAT AAT AAA GTT G*TA ATG AAC GAC TTG ATG ACG AAA – 3′ |
| attB2AtSYP111_Rv | 5′ – GGGG *AC CAC TTT GTA CAA GAA AGC TGG GT*T TCA AGA AGA GCT GAA ACT GGT – 3′ |
| attB3AtSYP112_Fw | 5′ – GGGG *ACA ACT TTG TAT AAT AAA GTT G*TA ATG AAT GAT CTG ATG ACA AAA – 3′ |
| attB2AtSYP112_Rv | 5′ – GGGG *AC CAC TTT GTA CAA GAA AGC TGG GT*T TCA ACG AGA AGC AAG CAT TGA – 3′ |
| attB3AtSYP122_Fw | 5′ – GGGG *ACA ACT TTG TAT AAT AAA GTT G*TA ATG AAC GAT CTT CTC TCC GGC – 3′ |
| attB2AtSYP122_Rv | 5′ – GGGG *AC CAC TTT GTA CAA GAA AGC TGG GT*T TTA GCG TAG TAG CCG CCG ATT – 3′ |
| attB3AtSYP123_Fw | 5′ – GGGG *ACA ACT TTG TAT AAT AAA GTT G*TA ATG AAC GAT CTT ATC TCA AGC – 3′ |
| attB2AtSYP123_Rv | 5′ – GGGG *AC CAC TTT GTA CAA GAA AGC TGG GT*T TCA AGG TCG AAG TAG AGT GTT – 3′ |
| attB3AtSYP124_Fw | 5′ – GGGG *ACA ACT TTG TAT AAT AAA GTT G*TA ATG AAT GAT TTA TTC TCT AGT – 3′ |
| attB2AtSYP124_Rv | 5′ – GGGG *AC CAC TTT GTA CAA GAA AGC TGG GT*T TCA CTT CAA CAT GAG CAT GAT – 3′ |
| attB3AtSYP125_Fw | 5′ – GGGG *ACA ACT TTG TAT AAT AAA GTT G*TA ATG AAC GAT TTA TTC TCT AAT – 3′ |
| attB2AtSYP125_Rv | 5′ – GGGG *AC CAC TTT GTA CAA GAA AGC TGG GT*T TCA CTT CAA CAT GAG CAT TAT – 3′ |
| attB3AtSYP131_Fw | 5′ – GGGG *ACA ACT TTG TAT AAT AAA GTT G*TA ATG AAC GAC CTC TTA AAG GGT – 3′ |
| attB2AtSYP131_Rv | 5′ – GGGG *AC CAC TTT GTA CAA GAA AGC TGG GT*T TTA GGC ACC ATT TTT CTG TGT – 3′ |
| attB3AtSYP132_Fw | 5′ – GGGG *ACA ACT TTG TAT AAT AAA GTT G*TA ATG AAC GAT CTT CTG AAG GGT TCG – 3′ |
| attB2AtSYP132_Rv | 5′ – GGGG *AC CAC TTT GTA CAA GAA AGC TGG GT*T TCA AGC ACT CTT GTT TTT CCA – 3′ |
| attB3AtSYP22_Fw | 5′ – GGGG *ACA ACT TTG TAT AAT AAA GTT G*TA ATG AGT TTT CAA GAT TTA GAA – 3′ |
| attB2AtSYP22_Rv | 5′ – GGGG *AC CAC TTT GTA CAA GAA AGC TGG GT*T TCA AGC TGC GAG TAC TAT AAT – 3′ |
| attB3AtSYP41_Fw | 5′ – GGGG *ACA ACT TTG TAT AAT AAA GTT G*TA ATG GCG ACG AGG AAT CGT ACG – 3′ |
| attB2AtSYP41_Rv | 5′ – GGGG *AC CAC TTT GTA CAA GAA AGC TGG GT*T TCA CAA GAA TAT TTC CTT GAG – 3′ |
| attB3AtSYP51_Fw | 5′ – GGGG *ACA ACT TTG TAT AAT AAA GTT G*TA ATG GCG TCT TCA TCG GAT TCA – 3′ |
| attB2AtSYP51_Rv | 5′ – GGGG *AC CAC TTT GTA CAA GAA AGC TGG GT*T TTA CAT ATA CTT AAC CAA CAT – 3′ |
| attB3AtSYP81_Fw | 5′ – GGGG *ACA ACT TTG TAT AAT AAA GTT G*TA ATG TCG AGA TTC AGA GAC AGG – 3′ |
| attB2AtSYP81_Rv | 5′ – GGGG *AC CAC TTT GTA CAA GAA AGC TGG GT*T TTA ACT GTA CCA ATC CAA GAA – 3′ |
| attB3AtVAMP721_Fw | 5′ – GGGG *ACA ACT TTG TAT AAT AAA GTT G*TA ATG GCG CAA CAA TCG TTG ATC – 3′ |
| attB2AtVAMP721_Rv | 5′ – GGGG *AC CAC TTT GTA CAA GAA AGC TGG GT*T TTA ACA CTT AAA CCC ATG GCA – 3′ |
| attB3AtVAMP722_Fw | 5′ – GGGG *ACA ACT TTG TAT AAT AAA GTT G*TA ATG GCG CAA CAA TCG TTG – 3′ |
| attB2AtVAMP722_Rv | 5′ – GGGG *AC CAC TTT GTA CAA GAA AGC TGG GT*T TTA TTT ACC GCA GTT GAA – 3′ |
| attB3ZmSYP121_Fw | 5′ – GGGG *ACA ACT TTG TAT AAT AAA GTT G*TA ATG AAC AGC CTG TTC TCG – 3′ |
| attB2ZmSYP121_Rv | 5′ – GGGG *AC CAC TTT GTA CAA GAA AGC TGG GT*T CTA GTT CTT ATT GAC GCC – 3′ |
| attB3NtSYP121_Fw | 5′ – GGGG *ACA ACT TTG TAT AAT AAA GTT G*TA ATG AAT GAT CTT TTT TCA – 3′ |
| attB2NtSYP121_Rv | 5′ – GGGG *AC CAC TTT GTA CAA GAA AGC TGG GT*T TCA TTT TTT CCA TGG CTG – 3′ |
| attB1AtPIP2;7_Fw | 5′ – GGGG *ACA AGT TTG TAC AAA AAA GCA GGC T*TA ATG TCG AAA GAA GTG AGC GAA – 3′ |
| attB4AtPIP2;7_Rv | 5′ – GGGG *AC AAC TTT GTA TAG AAA AGT TGG GT*G TTA ATT GGT TGC GTT GCT TCG – 3′ |
| attB1AtPIP2;7Δ1-13_Fw | 5′ – GGGG *ACA AGT TTG TAC AAA AAA GCA GGC T*TA ATG GGA AAA GAC TAC GTG GAT C – 3' |
| attB1AtPIP2;7Δ1-17_Fw | 5′ – GGGG *ACA AGT TTG TAC AAA AAA GCA GGC T*TA GTG GAT CCT CCA CCA GCT CCT – 3′ |
| attB1AtPIP2;7Δ1-23_Fw | 5′ – GGGG *ACA AGT TTG TAC AAA AAA GCA GGC T*TA CCA GCT CCT CTT CTC GAC ATG – 3′ |
| attB1AtPIP2;7Δ1-26_Fw | 5′ – GGGG *ACA AGT TTG TAC AAA AAA GCA GGC T*TA ATG GAC ATG GGT GAG CTC AAA TC – 3' |
| attB4PIP2;7-266Δ_Rv | 5′ – GGGG *AC AAC TTT GTA TAG AAA AGT TGG GT*G TTA TGA AGC TCT CAA TAT GTA – 3′ |
| attB4PIP2;7-270Δ_Rv | 5′ – GGGG *AC AAC TTT GTA TAG AAA AGT TGG GT*G TTA GGC CTT AAT TGC TGA AGC – 3′ |
| attB4PIP2;7-274Δ_Rv | 5′ – GGGG *AC AAC TTT GTA TAG AAA AGT TGG GT*G TTA GAA CGA GCC CAA GGC CTT – 3′ |
| Nt2;6][PIP2;7_Fw | 5′ – GAG CTC AAG AAG TGG TCC  TTC TAC AGA GCT CTC ATC – 3′ |
| Nt2;6][PIP2;7_Rv | 5′ – GAT GAG AGC TCT GTA GAA  GGA CCA CTT CTT GAG CTC – 3′ |
| PIP2;7][Ct2;6_Fw | 5′ – GCT TAC CAC CAA TAC ATA  TTG AGA GCT GGT GCA ATG – 3′ |
| PIP2;7][Ct2;6_Rv | 5′ – CAT TGC ACC AGC TCT CAA  TAT GTA TTG GTG GTA AGC – 3′ |
| Nt2;7][PIP2;6_Fw | 5′ – GAG CTC AAA TCC TGG TCT  TTC TAC AGA GCT GTC ATC – 3′ |
| Nt2;7][PIP2;6_Rv | 5′ – GAT GAC AGC TCT GTA GAA  AGA CCA GGA TTT GAG CTC – 3′ |
| PIP2;6][Ct2;7_Fw | 5′ – TTT TAC CAT CAG TTT GTG  TTG AGA GCT TCA GCA ATT – 3′ |
| PIP2;6][Ct2;7_Rv | 5′ – AAT TGC TGA AGC TCT CAA  CAC AAA CTG ATG GTA AAA – 3′ |
| PIP2;7-D][TM5-PIP2;6_Fw | 5′ – AGC GCT CGT GAC TCT CAC ATC  CCT GTA TTA GCA CCA TTG CCA – 3′ |
| PIP2;7-D][TM5-PIP2;6_Rv | 5′ – TGG CAA TGG TGC TAA TAC AGG  GAT GTG AGA GTC ACG AGC GCT – 3′ |
| PIP2;7-C][TM4-PIP2;6_Fw | 5′ – GTA GCT GAC GGT TAC AGC AAA  GGC GTT GGG GTT GGT GCT GAG – 3′ |
| PIP2;7-C][TM4-PIP2;6_Rv | 5′ – CTC AGC ACC AAC CCC AAC GCC  TTT GCT GTA ACC GTC AGC TAC – 3′ |
| PIP2;7-TM3][C-PIP2;6_Fw | 5′ – GTG GGT TTC GTG AAA GCT TTC  CAG TCG ACT TAT TAC AAC CGC – 3′ |
| PIP2;7-TM3][C-PIP2;6_Rv | 5′ – GCG GTT GTA ATA AGT CGA CTG  GAA AGC TTT CAC GAA ACC CAC – 3′ |
| PIP2;7-B][TM3-PIP2;6_Fw | 5′ – TTC TTG GCC CGT AAG GTC TCT  TTG GTT AGA GCT GTG TCG TAC – 3′ |
| PIP2;7-B][TM3-PIP2;6_Rv | 5′ – GTA CGA CAC AGC TCT AAC CAA  AGA GAC CTT ACG GGC CAA GAA – 3′ |
| attB1AtPIP2;1_Fw | 5′ – GGGG *ACA AGT TTG TAC AAA AAA GCA GGC T*TA ATG GCA AAG GAT GTG GAA GCC – 3′ |
| attB4AtPIP2;1_Rv | 5′ – GGGG *AC AAC TTT GTA TAG AAA AGT TGG GT*G TTA GAC GTT GGC AGC ACT TCT – 3' |
| attB1AtPIP2;2_Fw | 5′ – GGGG *ACA AGT TTG TAC AAA AAA GCA GGC T*TA ATG GCC AAA GAC GTG GAA GGA – 3′ |
| attB4AtPIP2;2_Rv | 5′ – GGGG *AC AAC TTT GTA TAG AAA AGT TGG GT*G TCA AAC GTT GGC TGC ACT TCT – 3′ |
| attB1AtPIP2;3_Fw | 5′ – GGGG *ACA AGT TTG TAC AAA AAA GCA GGC T*TA ATG GCT AAA GAC GTG GAA GGA – 3′ |
| attB4AtPIP2;3_Rv | 5′ – GGGG *AC AAC TTT GTA TAG AAA AGT TGG GT*G TTA AAC GTT GGC TGC ACT TCT – 3′ |
| attB1AtPIP2;4_Fw | 5′ – GGGG *ACA AGT TTG TAC AAA AAA GCA GGC T*TA ATG GCA AAA GAC TTG GAT GTG – 3′ |
| attB4AtPIP2;4_Rv | 5′ – GGGG *AC AAC TTT GTA TAG AAA AGT TGG GT*G TTA AGC AAA GCT CCT AAA GGA – 3′ |
| attB1AtPIP2;5_Fw | 5′ – GGGG *ACA AGT TTG TAC AAA AAA GCA GGC T*TA ATG ACG AAG GAA GTG GTT GGT– 3′ |
| attB4AtPIP2;5_Rv | 5′ – GGGG *AC AAC TTT GTA TAG AAA AGT TGG GT*G TTA AAC GTG AGG CTG GCT CCT – 3′ |
| attB1AtPIP2;6_Fw | 5′ – GGGG *ACA AGT TTG TAC AAA AAA GCA GGC T*TA ATG ACG AAG GAT GAG TTG ACG – 3′ |
| attB4AtPIP2;6_Rv | 5′ – GGGG *AC AAC TTT GTA TAG AAA AGT TGG GT*G TTA AGC ATG GAG CTC ATG AAG – 3′ |
| attB1AtPIP2;8_Fw | 5′ – GGGG *ACA AGT TTG TAC AAA AAA GCA GGC T*TA ATG TCA AAA GAA GTG AGT GAA – 3′ |
| attB4AtPIP2;8_Rv | 5′ – GGGG *AC AAC TTT GTA TAG AAA AGT TGG GT*G TCA ATT GGT TGG GTT GCT GCG – 3′ |
| attB1AtPIP1;4_Fw | 5′ – GGGG *ACA AGT TTG TAC AAA AAA GCA GGC T*TA ATG GAA GGC AAA GAA GAA GAT – 3′ |
| attB4AtPIP1;4_Rv | 5′ – GGGG *AC AAC TTT GTA TAG AAA AGT TGG GT*G CTA ACT CTT GCT CTT GAA AGG – 3′ |
